# Immunomodulatory microneedles restore mitochondrial homeostasis and balance TGF-β/TNF-α signaling to accelerate diabetic wound repair

**DOI:** 10.1016/j.mtbio.2025.102687

**Published:** 2025-12-14

**Authors:** Minjian Liao, Xinmin Guo, Longbao Feng, Qing Peng, Jianhao Liang, Aleh Kuzniatsou, Rui Guo, Pan Yu, Shuqin Zhou

**Affiliations:** a Key Laboratory of Biomaterials of Guangdong Higher Education Institutes, Key Laboratory of Regenerative Medicine of Ministry of Education, Guangdong Provincial Engineering and Technological Research Centre for Drug Carrier Development, Department of Biomedical Engineering, Jinan University, Guangzhou, 510632, China; b Department of Ultrasound, Guangzhou Red Cross Hospital of Jinan University, Guangzhou, 510220, China; c Central Laboratory of The Second Affiliated Hospital, School of Medicine, The Chinese University of Hong Kong, Shenzhen & Longgang District People's Hospital of Shenzhen, Shenzhen, 518172, China; d Institute of Biochemistry of Biologically Active Compounds of the National Academy of Sciences of Belarus, Grodno, Belarus; e Department of Burn and Plastic Surgery, Jinling Hospital, Affiliated Hospital of Medical School, Nanjing University, Nanjing, China; f Department of Anesthesiology of The Second Affiliated Hospital, School of Medicine, The Chinese University of Hong Kong, Shenzhen & Longgang District People's Hospital of Shenzhen, Shenzhen, 518172, China; g Department of Anesthesiology, Zhujiang Hospital, Southern Medical University, Guangzhou, 510000, China

**Keywords:** MXene@Zn-MOF, Dual-layer microneedles, TGF-β signaling pathways, Diabetic wound

## Abstract

Diabetic infected wounds bring great physical and psychological burden to patients. To solve this problem, removing bacteria and regulating the local immune microenvironment have become effective measures. However, traditional wound repair materials are difficult to break through the bacterial biofilm covering the wound site and thus cannot effectively regulate the internal immune environment of the wound. A bilayer multifunctional sulfonated chitosan (SCS)/methacrylated hyaluronic acid (HAMA)/methacrylated collagen III (Col_III_MA) (SCS/HAMA/Col_III_MA, SHC) microneedle loaded with MXene@Zn-MOF (MXZ) composite was designed for diabetic infected wound repair. The MXZ composite achieved more than 99 % bacterial inhibition against both *E. coli* and *S. aureus*, and was able to effectively remove the bacterial biofilm. In addition, SHC + MXZ microneedles were able to promote angiogenesis by activating the Transforming Growth Factor-β (TGF-β) signaling pathway. It also regulated macrophage polarization towards the M2 phenotype and inhibited the Tumor Necrosis Factor-α (TNF-α) signaling pathway to reduce the level of inflammation. Further studies on mitochondrial membrane potential showed that SHC + MXZ microneedles were able to restore the decrease in mitochondrial membrane potential caused by ROS and then restore mitochondrial function. In diabetic infected wound repair results showed that SHC + MXZ microneedles effectively promoted diabetic infected wound repair by promoting angiogenesis and reducing the inflammation level of the wound tissue. Finally, the regulation of related gene expression was explored by transcriptome sequencing, and the intrinsic mechanism of SHC MXZ microneedles in promoting the repair of diabetic infected wounds by regulating the TGF-β and TNF-α signaling pathways was elucidated.

## Graphical abstract

**Figure:**
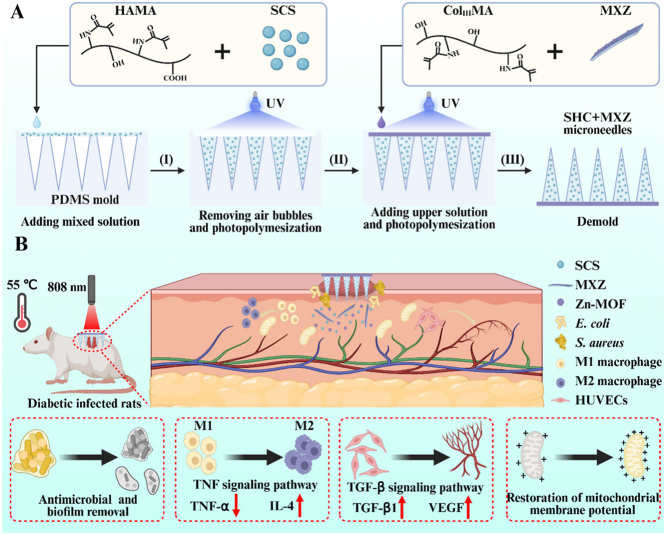

## 1 Introduction

Diabetes has become a global metabolic disease, and according to the International Diabetes Federation's 2021 statistics, the cumulative number of patients worldwide has reached 537 million, resulting in a huge public health burden [1,2]. Its prolonged high-glucose environment leads to vasculopathy and immune dysfunction, which in turn leads to a variety of serious complications [3]. In particular, chronic diabetic wounds provide ample nutrients for pathogens due to the local high-glucose microenvironment, which in turn severely weakens host defenses and immune responses [4]. Some patients may develop infections and form bacterial biofilms that are difficult to remove, eventually developing into chronic wounds that are difficult to heal [5]. These wounds are characterized by complex treatment, low cure rate, high recurrence rate and high cost, which has become a major problem in clinical treatment [6]. Current clinical treatment is mainly through surgical debridement combined with antibiotic therapy, although the infection can be temporarily controlled [7]. However, it cannot effectively restore the dysregulation of the immune microenvironment caused by high-glucose, and the obstacle of vascular regeneration still exists [8]. This ultimately leads to stagnation of wound repair in long-term inflammation. Therefore, to address the two major problems of infection and immune microenvironment dysregulation in diabetic infected wounds. The development of new therapeutic materials with both efficient antimicrobial and microenvironment regulation functions has important clinical needs and application value [9].

To address the challenges of repairing diabetic infected wounds, it is crucial to develop repair materials that combine efficient antimicrobial and immune microenvironmental modulation functions. MXene (two-dimensional transition metal carbon nitride) has become an emerging material for antibacterial therapy due to its efficient photothermal properties and excellent biocompatibility [10]. It can be rapidly heated up under the irradiation of near-infrared ray (808 nm), and physically destroys the structure of bacterial membrane and degrades the extracellular polysaccharide of biofilm [11]. It avoids the problem of bacterial resistance caused by traditional antibiotic treatment and achieves efficient removal of bacteria and bacterial biofilm [12,13]. However, a single photothermal antimicrobial is difficult to reverse the immune microenvironmental dysregulation in diabetic wounds and cannot effectively address immune cell dysfunction and impaired vascular regeneration. Metal-organic framework (MOF) materials have shown unique advantages in the field of wound repair due to their environmentally responsive degradation properties [14]. However, most MOFs still rely on exogenous drug loading and delivery, whereas Zn-MOF can directly act as a key signal for immunomodulation due to its unique acid-responsive Zn^2+^ release properties and biocompatibility [15]. Significantly inhibited the TNF-α signaling pathway by activating macrophage M2-type polarization [16]. In addition, Zn^2+^ can directly activate the angiogenesis-related TGF-β signaling pathway to promote angiogenesis and VEGF secretion [17]. Therefore, by constructing the MXene@Zn-MOF (MXZ) composite, a cascade reaction system integrating antibacterial activity and immunomodulation is established. The photothermal effect of MXene can rapidly eliminate bacteria on the wound surface and disrupt the physical barrier of bacterial biofilms. Furthermore, the photothermal heating and the local acidic microenvironment of the wound synergistically trigger the accelerated degradation of Zn-MOF, providing a rapid release pathway for subsequent Zn^2+^ ions. Subsequently, Zn^2+^ ions inhibit key pro-inflammatory signaling pathways to reduce inflammation and drive angiogenesis, forming a functionally complementary organic whole. However, how to efficiently deliver this material to the deep tissue covered by the biofilm requires the design of new delivery systems to break through this barrier.

Microneedles, a drug delivery platform consisting of multiple micro needles, have shown great advantages in the field of diabetic wound repair [18,19]. Traditional diabetic wound repair materials, such as hydrogels and nanofiber membranes, can accelerate wound healing but suffer from drawbacks such as poor drug penetration and low treatment efficiency [20]. Compared to conventional wound repair materials, microneedles can easily penetrate the wound barrier, facilitating deep drug delivery [21]. In addition, penetration of the chronic wound barrier creates hundreds of micron-sized holes and provides mechanical stimulation, thereby creating a rapid pathway for therapeutic agents [22]. In particular, hydrogel-based microneedles can spontaneously dissolve and release drugs after deep drug delivery, providing unique application advantages in chronic wound repair and tissue regeneration [23]. Although conventional hydrogel microneedles have achieved certain progress in drug delivery, most of them suffer from relatively single functionality and fail to adapt to the complex, temporally dynamic pathological microenvironment of diabetic infected wounds [24]. In contrast, bilayer microneedles can realize programmed sequential release through deliberate structural design: the tip layer rapidly penetrates the wound barrier, exerts immediate antibacterial action, and eliminates bacterial biofilms upon dissolution, whereas the base layer provides sustained regulation of the immune microenvironment and promotes long-term tissue regeneration [25]. This realizes a leap from a single function to time-sequential regulation, thereby holding great promise for systematically addressing multiple obstacles in the repair of diabetic infected wounds.

**Scheme 1:**
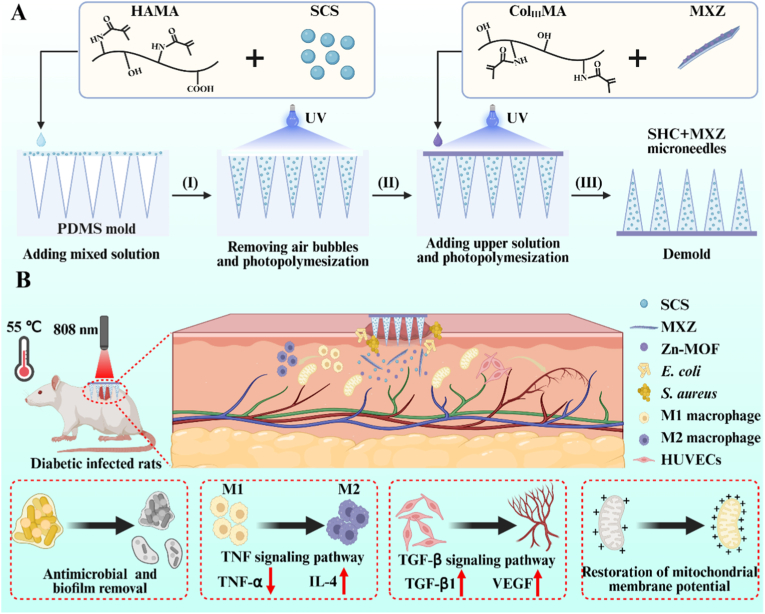
**Preparation of microneedles and diabetic infected wound repair.** (A) Preparation of the microneedles; (B) Microneedles to promote diabetic infected wound repair.

This study addressed the pathological challenges in diabetic infected wounds, including bacterial biofilm colonization, persistent inflammation, impaired vascular regeneration, and mitochondrial dysfunction. We innovatively constructed a MXene@Zn-MOF (MXZ) composite and loaded it into a bilayer hydrogel microneedle (SHC) system to achieve a precise unified therapeutic strategy (Scheme 1). The needle tip layer rapidly penetrated and disrupted biofilms, enabling biofilm clearance through the photothermal effect of MXene. The base layer continuously released Zn^2+^, which suppressed the TNF-α pro-inflammatory pathway to reduce inflammation levels, while simultaneously activating the TGF-β/Smad3 signaling axis to promote vascular regeneration and reversed the ROS-induced decrease in mitochondrial membrane potential, thereby comprehensively reprogramming the immune microenvironment. Animal experiments demonstrated that this microneedle significantly accelerates the healing of diabetic infected wounds, enhanced collagen deposition, and promoted vascular regeneration. Transcriptomic sequencing further revealed the molecular mechanism by which it drives staged healing through the synergistic regulation of the TNF-α and TGF-β signaling pathways. By simultaneously addressing the three major challenges of infection control, immune microenvironment remodeling, and tissue regeneration, this approached significantly accelerates the entire repair process of diabetic wounds.

## 2 Result and discussion

### 2.1 Preparation and characterization of MXZ and microneedles

To confirm the synthesis of human recombinant collagen methacryloyl (Col_III_MA), we analyzed the changes in recombinant human recombinant collagen (Col_III_) before and after modification by ^1^H NMR spectroscopy. The results were shown in Fig. S1 A, compared with Col, Col_III_MA showed two new absorption peaks at δ = 5.30 and 5.54, which corresponded to the characteristic peaks of the methylene hydrogen resonance of MA [26], indicating the successful synthesis of Col_III_MA. Hyaluronic acid methacryloyl (HAMA) was also analyzed by ^1^H NMR spectroscopy and the results were shown in Fig. S1 B. Compared to hyaluronic acid (HA), a new absorption peak at δ = 5.78 corresponded to the methylene hydrogen resonance of MA. In addition, a new absorption peak also appeared between δ = 1.00–1.25, which corresponded to the characteristic peak of methyl hydrogen resonance on MA [27], further indicating the successful synthesis of HAMA. The synthesis of sulfonated chitosan (SCS) was subsequently verified by FT-IR, and we verified the absorption peaks of SCS by FT-IR, and the results were shown in Fig. S1 C. Compared with CS, the new characteristic peaks at 811 and 1225 cm^−1^ correspond to C-O-S stretching vibration and S=O telescoping vibration [28], which were mainly present in the sulphonic acid moiety, and the above results proved that SCS was successfully obtained. Subsequently, we synthesized the metal-organic framework Zn-MOF at room temperature and confirmed its structure by XRD, and the results were shown in Fig. S1 D. Absorption peaks at 2θ = 7.2, 10.2, 12.6, 14.6, 16.3, and 17.9, which correspond to the (0 1 1), (0 0 2), (1 1 2), (0 2 2), (0 1 3), and (2 2 2) crystallographic planes [29] and correspond to the standard card No. 00-062-1030, proved the structure of Zn-MOF. Further we determined the XRD of MXene and the results were shown in Fig. S1 E. The absorption peaks at 2θ = 7.1, 12.8, 33.8, 39.3, 53.0, and 60.6 correspond to (0 0 2), (0 0 4), (1 0 1), (1 0 4), (1 1 0), and (1 1 2) crystal planes, respectively [30]. And the absorption peaks belonging to Zn-MOF clearly appeared after combining with Zn-MOF to form MXZ, which proved the successful combination of Zn-MOF and MXene. We confirmed the binding mode of Zn-MOF and MXene by Zeta potential detection, and the results were shown in Fig. S1 F. Zn-MOF and MXene exhibited positive and negative electrical properties, respectively, and the Zeta potential of MXZ after binding exhibited a negative charge but was significantly larger than that of MXene, which proved that the two could be effectively bound by electrostatic interactions. Finally, we observed its microstructure by SEM and mapping, and the results were shown in Fig. S1 G. It showed a clear multilayer structure before peeling, and a single layer of MXene was formed after ultrasonic and centrifugal peeling, and Zn-MOF was clearly observed to appear on its surface after the formation of MXZ. And Zn element also appeared in the elemental scan, which further verified the formation of MXZ.

It had been shown that MXene had good photothermal conversion ability and could efficiently convert NIR light energy into thermal energy [31]. Therefore, we explored the photothermal effect of the prepared MXZ. The thermograms of different concentrations of MXZ at different times under 808 nm NIR irradiation were monitored in real time using a thermal imager, and the results were shown in Fig. S2 A. The temperature rise curves of different concentrations of MXZ (0, 0.25, 0.50, 0.75 and 1.00 mg/mL) were recorded by a thermocouple thermometer, and the results were shown in Fig. S2 B. Since MXZ could effectively absorb light energy and convert it into heat energy, the temperature could be rapidly increased to 65 °C within 10 min. Fig. S2. C showed the warming effect of MXZ recorded by varying the power of NIR (0.5, 1.0, 1.5 and 2.0 W/cm^2^), from which it was clear that the maximum temperature increased to 78 °C with increasing power, which played an important role in killing the bacteria. To evaluate the photothermal stability of MXZ, we performed five heating and cooling cycles. After five cycles, there was no significant decrease in the maximum temperature (Fig. S2 D), showing good photothermal stability. Finally, according to the reported photothermal efficiency calculation method [32], we calculated the photothermal efficiency of MXZ, and the results were shown in Fig. S2 E-F. The photothermal conversion efficiency of MXZ was obtained to be 32.96 %, which had excellent photothermal performance and could effectively use high temperature to remove bacterial biofilm.

**Fig. 1:**
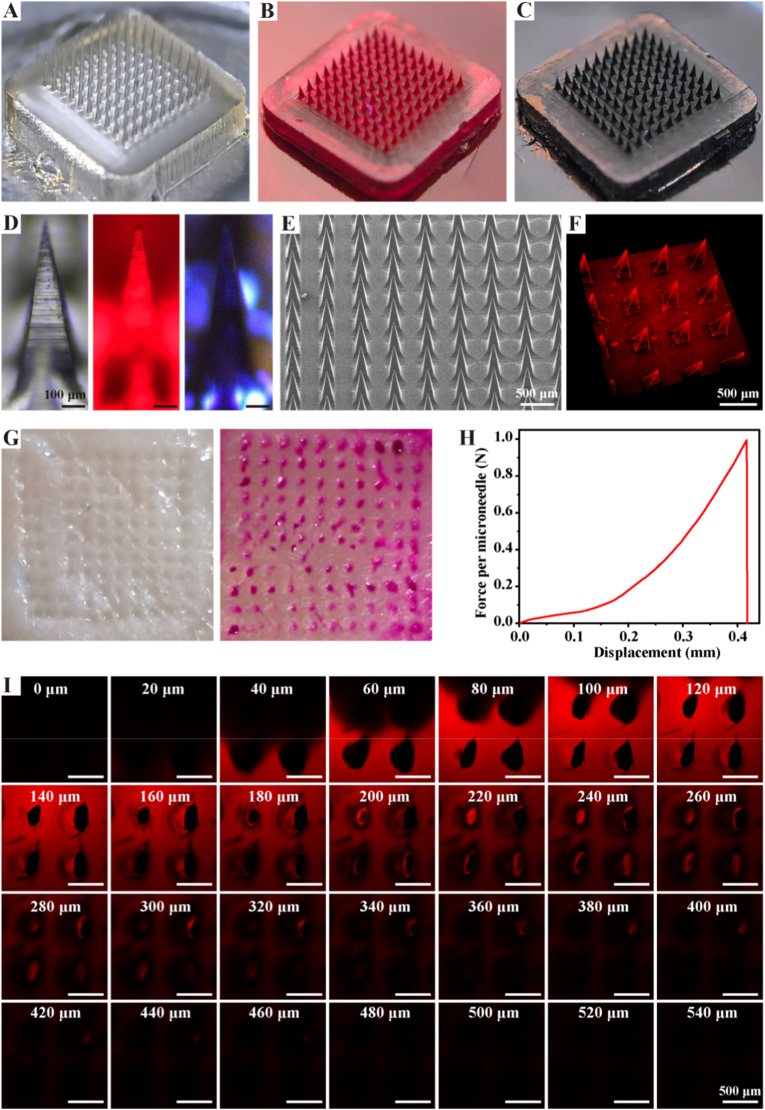
**Structural characterization of microneedles**. A. Microneedle Physical Drawing; B. Physical drawing of microneedle with adriamycin added; C. Physical drawing of microneedle with methylene blue added; D. Optical microscope view of microneedle tip; E. SEM image of microneedle; F. Three-dimensional diagram of microneedles; G. Schematic diagram of microneedle piercing pig skin; H. The compression curve of the microneedle; I. Microneedle puncture depth of agarose.

In recent developments, microneedles were used for deep drug delivery to promote chronic wound repair due to their good penetration ability to cross the skin stratum corneum barrier [33]. Therefore, we constructed a double-layer microneedle using HAMA, SCS, and Col_III_MA as materials. We observed the structure of the microneedles through a macro camera, and the results were shown in Fig. 1 A-C, where uniform tip structures were observed. Observation of a single tip under a light microscope (Fig. 1. D) showed a complete tip. In addition, the structure of the microneedles was observed under SEM and the results were shown in Fig. 1. E. Each needle tip showed a conical shape with a height of 800 μm and a spacing of 200 μm at the bottom. The three-dimensional structure of the microneedles was subsequently observed using confocal fluorescence microscopy (Fig. 1. F). To verify the piercing effect of microneedles, we used microneedles to pierce fresh pig skin, and the results were shown in Fig. 1. G. Orderly pinholes appeared on the pig skin after microneedle puncture, and the pinholes were more obvious after adding dye. The mechanical strength of the needle tip could effectively ensure that the microneedle could effectively pierce the skin, which was evaluated by compression test. The results were shown in Fig. 1 H. The mechanical strength of the needle tip reached 1.0 N/needle, which was considerably more than the reported force required to penetrate the skin (0.045 N/needle) [34]. Finally, we explored the piercing depth of the microneedle by confocal fluorescence microscopy, and the results were shown in Fig. 1 I. The microneedles were able to puncture to a depth of 440 μm under the skin to deliver drugs to deep tissues, showing excellent deep drug delivery capability.

### 2.2 Evaluation of antibacterial effect *in vitro*

**Fig. 2:**
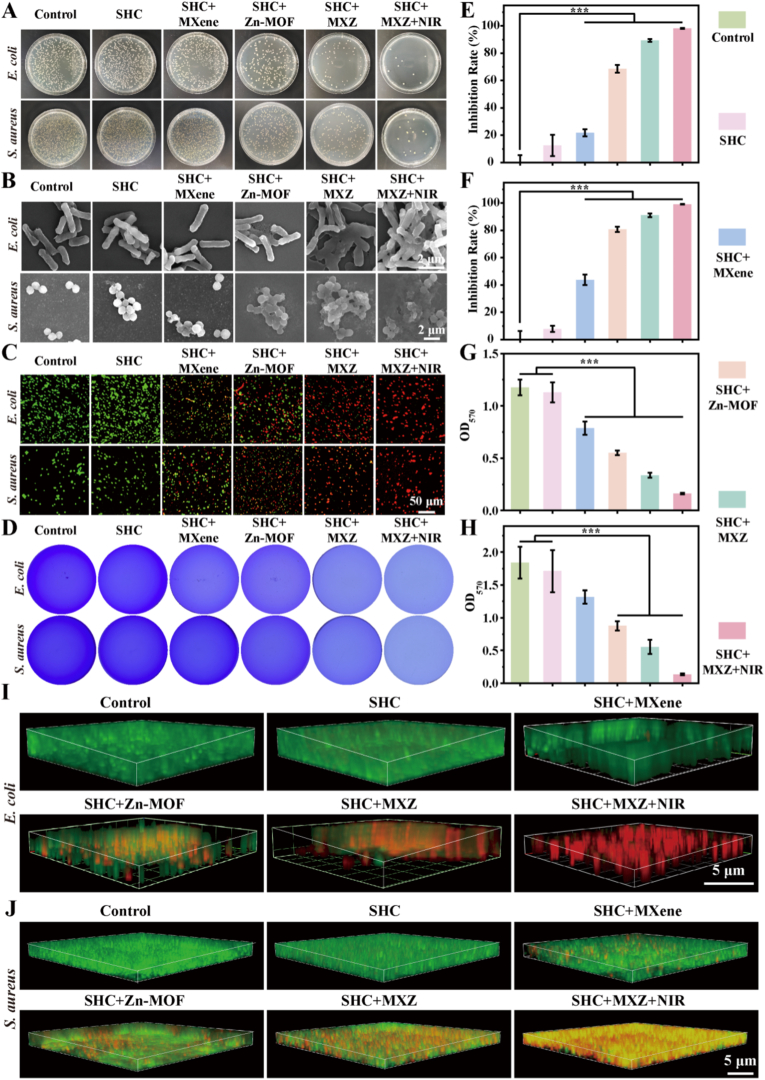
***In vitro* antimicrobial characterization of microneedles.** A. Number of colonies of *E. coli* and *S. aureus* on agar plates; B. SEM images of *E. coli* and *S. aureus*; C. Bacterial live-dead staining of *E. coli* and *S. aureus*; D. Crystal violet staining of bacterial biofilms of *E. coli* and *S. aureus*; E. Bacterial inhibition rate of *E. coli*; F. Bacterial inhibition rate of *S. aureus*; G. Absorbance of *E. coli* bacterial biofilm at 570 nm; H. Absorbance of *S. aureus* bacterial biofilm at 570 nm; I. Live-dead staining of *E. coli* bacterial biofilm; J. Live-dead staining of *S. aureus* bacterial biofilm.

Chronic wound repair process is susceptible to bacterial infection, especially bacterial biofilm localization, which exacerbated the level of inflammation at the wound site to inhibit wound healing [35]. Therefore, antimicrobial and bacterial biofilm removal played an important role in the process of wound repair. We selected Gram-negative *Escherichia coli* (*E. coli*) and Gram-positive *Staphylococcus aureus* (*S. aureus*), which were commonly found in wound infections. First, the antibacterial effects of MXZ at different concentrations on *E. coli* and *S. aureus* were explored. The results of the agar plate images were shown in Fig. S3 A and B, these results indicated that as the concentration of MXZ increased, the number of bacterial colonies on the plates decreased, showing a concentration-dependent characteristic. After quantitative statistical analysis of the number of bacterial colonies, the results are presented in Fig. S3 C and D, and the antibacterial rates also exhibited a similar concentration-dependent trend. Subsequently, the co-cultivation method was used to evaluate the antibacterial efficacy of the microneedles, and the results are shown in Fig. 2 A, E, and F. The microneedles loaded with MXZ microneedles showed a certain antibacterial effect, with inhibition rates of 89.3 % and 91.1 % against *E. coli* and *S. aureus*, respectively, which was attributed to the binding of Zn ions in MXZ to the bacterial cell membrane, which interfered with bacterial protein synthesis upon entry [36]. In addition, after NIR irradiation, the number of colonies decreased significantly and the inhibition rates of *E. coli* and *S. aureus* increased to 98.2 % and 99.2 %, which was mainly attributed to the increase in temperature after NIR irradiation that led to further bacterial death. Further changes in bacterial morphology were observed by SEM (Fig. 2. B), significant changes in bacterial morphology were observed in the SHC + MXZ and SHC + MXZ + NIR groups when compared to the control group. The cell membrane surface was damaged and the bacterial contents with protein efflux resulted in bacterial death. Subsequently, the viability of the bacteria was assessed by bacterial live-dead staining, and the results were shown in Fig. 2. C. The field of view of the control group was basically green fluorescence, indicating that the bacteria survived in large numbers. In contrast, the SHC + MXZ and SHC + MXZ + NIR groups rarely showed green fluorescence within the field of view, and most of them showed red fluorescence, indicating bacterial death, which was consistent with the above results. We further investigated the effect of bacterial biofilm removal, the dissociation of crystalline violet dye produces positively charged colored ions that bind to the bacterial biofilm and had a significant absorbance at 570 nm [37]. The results of crystalline violet staining were shown in Fig. 2 D, G and H. The control group had the darkest color and the SHC + MXZ and SHC + MXZ + NIR groups had lighter color. The quantitative results also showed that the absorbance at 570 nm of the SHC + MXZ and SHC + MXZ + NIR groups was significantly smaller than that of the control group, showing good biofilm removal. Finally, to directly show the destructive effect of microneedles on the biofilm, we assessed the survival of the biofilm by biofilm live-dead staining and observed the change in the thickness of the biofilm by confocal fluorescence microscopy. The results were shown in Fig. 2 I, J. After SHC + MXZ and NIR treatments, the thickness of bacterial biofilm decreased, and the *E. coli* biofilm was obviously broken. Especially after NIR treatment, the red fluorescence was significantly enhanced, indicating that the aggregated biofilms were all in a dead state. This is mainly attributed to the photothermal conversion ability of MXZ, which converted light energy into heat energy, causing the local temperature of the bacterial biofilm to rise. The integrity of the bacterial biofilm, which consisted of polysaccharides and bacterial secreted proteins, was destroyed at high temperatures. In addition, high temperature disrupted the activity of bacterial metabolic enzymes, and the reduction of secreted proteins reduced the thickness of bacterial biofilm. Photothermal removal of bacterial biofilms provided an efficient and non-resistant method and had become an important strategy to combat recalcitrant bacterial biofilms.

### 2.3 Biocompatibility and pro-angiogenesis of microneedles *in vitro*

**Fig. 3:**
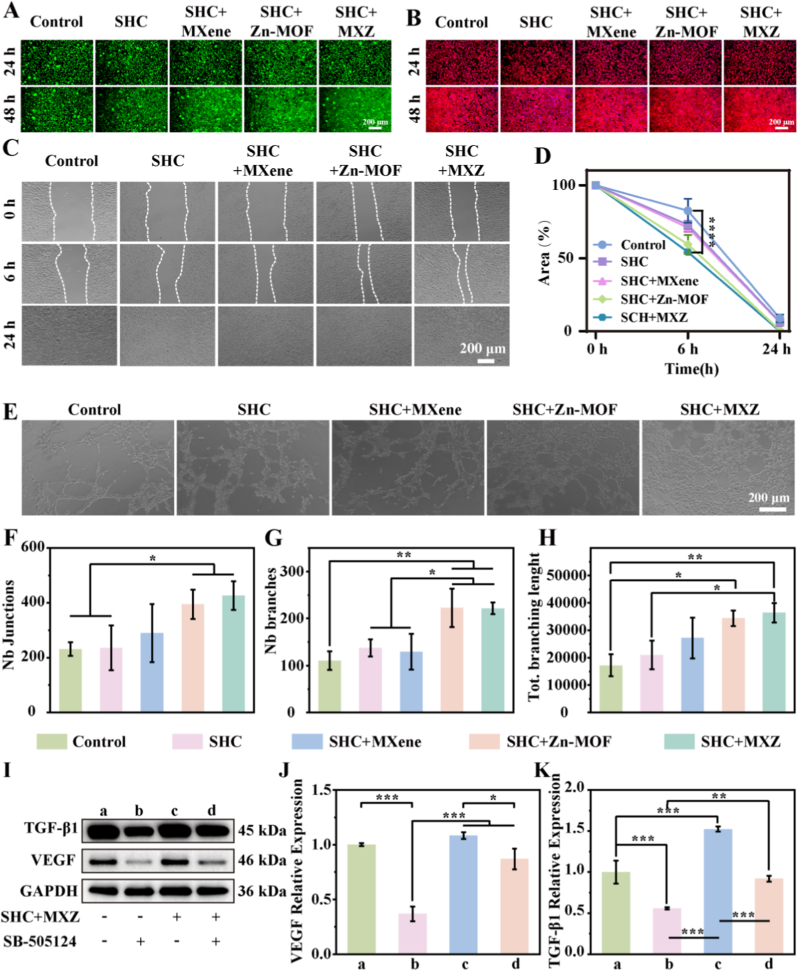
**Biocompatibility, tubulation and migration of microneedles.** A. Live-dead staining of microneedles; B. Cytoskeletal staining of microneedles; C. Microneedle-promoted L929 cell migration images; D. L929 cell scratch area closure rate; E. Image of microneedles promoting angiogenesis; F. Number of junctions of microneedles promoting angiogenesis; G. Number of branches of microneedles promoting angiogenesis; H. Total branching length of microneedle promoting angiogenesis; I. Verify the TGF-β signaling pathway by Western Blot (WB); Quantitative statistics of the qPCR of TGF-β1 (J) and VEGF (K).

Good biocompatibility is essential to prevent adverse effects of wound repair materials on tissues and cells [38]. To assess whether MXZ causes toxicity in normal tissue cells, we examined the cell viability of different concentrations of MXZ co-cultured with L929 cells for 24 h. The results were shown in Fig. S4 A. There was no significant toxicity to the cells when the concentration was lower than 1 mg/mL, and the cell viability was significantly reduced when the concentration was increased to more than 2.5 mg/mL. We further evaluated the effect of different concentrations of MXZ on L929 cell apoptosis by flow cytometry. As shown in Fig. S5 A, no significant apoptosis was observed when the MXZ concentration was increased up to 1 mg/mL. However, when the concentration exceeded 2.5 mg/mL, apparent apoptosis began to occur. Quantitative analysis of the apoptosis rate further confirmed this result (Fig. S5 C). Therefore, we chose a concentration of 1 mg/mL of MXZ for subsequent experiments. We also tested the biocompatibility of the microneedles, and the results were shown in Fig. S4 B. The results showed that cell viability was not affected by incubating L929 cells with different microneedle immersion solutions for 24, 48, and 72 h. We also assessed the effects of different microneedles on L929 cell apoptosis by flow cytometry. As shown in Fig. S5 B, D, all microneedle groups demonstrated favorable biocompatibility, with results comparable to the control group and no significant differences observed. Further we evaluated the effect of microneedles on L929 cells by cell live-dead staining and skeleton staining, and the results were shown in Fig. 3 A, B. No red color appeared in the field of view, indicating that the cells did not appear dead, reflecting good biocompatibility. Subsequently we evaluated the ability of SHC + MXZ microneedles to promote cell migration by cell scratch assay, and the results were shown in Fig. 3 C, D. The SHC + MXZ microneedle extract promoted the migration of L929 cells, and the area of the scratch was reduced to 55.6 % at h 6, which was significantly lower than that of the control group (79.7 %), suggesting that SHC + MXZ microneedles possessed the property of promoting cell migration and healing. Impaired angiogenesis is a major obstacle facing the chronic wound repair process, therefore, promoting angiogenesis is crucial for chronic wound repair [39]. We evaluated the ability of SHC + MXZ microneedles to promote angiogenesis *in vitro* by co-culturing HUVEC with SHC + MXZ microneedle extract on Matrigel for 4 h. The results were shown in Fig. 3. E. Significant tube formation was seen in the SHC + MXZ group compared to the control group. Further quantification of the total number of connections, total number of branches and total branch length results were shown in Fig. 3 F-H. The total number of connections, total number of branches and total branch length of SHC + Zn-MOF and SHC + MXZ groups were significantly higher than that of the control group, which was consistent with our observations. In addition, we detected the expression levels of angiogenesis-related factors TGF-β1 and VEGF by ELISA, and the results were shown in Fig. S6. The expression levels of TGF-β1 and VEGF were relatively low in the control group. In contrast, their expression was significantly increased in the SHC + MXZ group, indicating a promotion effect on angiogenesis. This result was also consistent with the aforementioned findings. It has been reported that the TGF-β1 signaling pathway promoted VEGF expression through activation of the Smad3-VEGF signaling axis, which further promoted angiogenesis [40]. We further investigated whether SHC + MXZ promoted VEGF expression through the TGF-β1 signaling pathway, and the results were shown in Fig. 3 I. SB-505124 was a selective inhibitor of the TGF-β1 receptor [41], significantly suppressed the expression of TGF-β1 and VEGF in the presence of the SB-505124 inhibitor, and the uninhibited SHC + MXZ group had higher TGF-β1 and VEGF expression than the control group. In addition, TGF-β1 and VEGF expression enhanced by SHC + MXZ was also significantly attenuated in the presence of SB-505124 inhibitor. Finally, we also quantified the relative expression of TGF-β1 and VEGF by qPCR, and the results were shown in Fig. 3 J, K. The above results suggested that SHC + MXZ promoted VEGF transcription mainly by activating the Smad3-VEGF signaling axis of the TGF-β1 signaling pathway, which promoted the expression of VEGF and TGF-β1, and thus promoted angiogenesis. In summary, the results of our in *vitro* experiments indicated that SHC + MXZ microneedles could significantly enhance the tube formation ability of HUVECs. More importantly, verified by Western Blot, qPCR, and experiments with the TGF-β receptor inhibitor SB-505124, we confirmed that this pro-angiogenic effect was mainly attributed to the activation of the TGF-β1/Smad3 signaling pathway driven by the sustained release of Zn^2+^ ions from MXZ. The activated TGF-β1/Smad3 axis further upregulated the expression of its key downstream factor, vascular endothelial growth factor (VEGF), thereby forming an effective positive regulatory loop that promoted angiogenesis. This provided a clear molecular mechanism for the role of MXZ composites in addressing angiogenesis disorders in diabetic wounds.

### 2.4 Anti-inflammatory, ROS scavenging and mitochondrial membrane potential staining *in vitro*

**Fig. 4:**
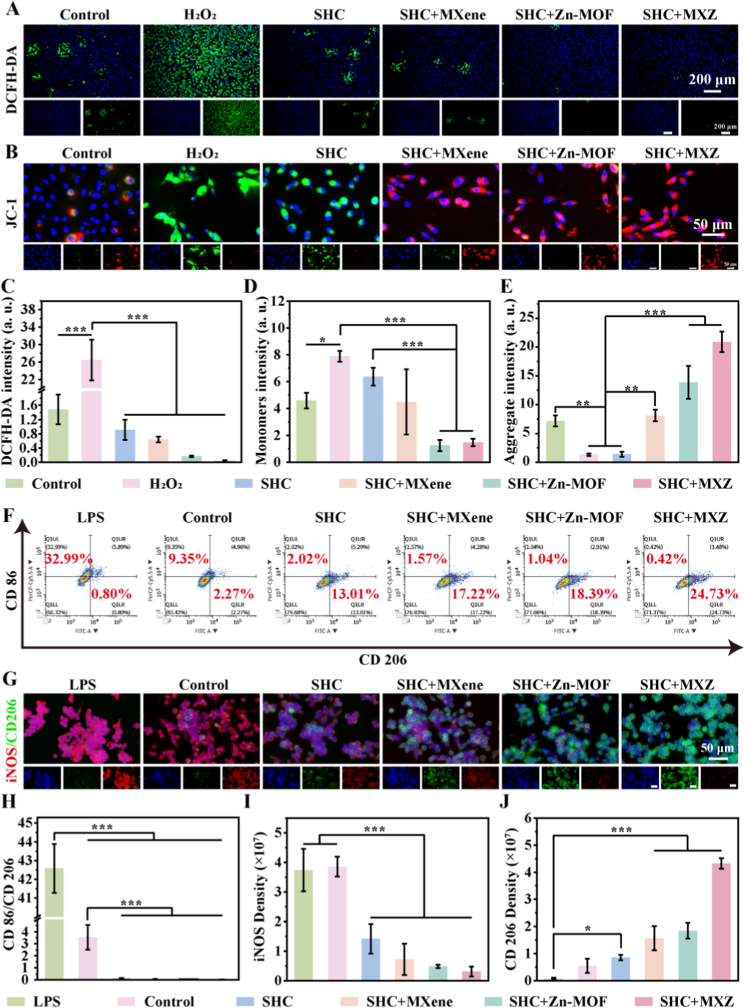
**Anti-inflammatory, ROS scavenging and mitochondrial membrane potential staining *in vitro*.** A. Microneedle clearance of ROS produced by L929 cells; B. Immunofluorescence staining of mitochondrial membrane potential of microtargeted L929 cells; C. Quantitative statistics of ROS fluorescence intensity; D. Quantitative statistics of monomers fluorescence intensity; E. Quantitative statistics of aggregate fluorescence intensity; F. Flow cytometry to assess the anti-inflammatory properties of microneedles; G. Immunofluorescence staining of CD206 and iNOS; H. The ratio of CD86/CD206; I. iNOS quantitative fluorescence intensity statistics; J. CD206 quantitative fluorescence intensity statistics.

The accumulation of ROS at the wound site was prone to trigger adverse effects such as cellular senescence and increased inflammation [42]. We stimulated L929 cells by H_2_O_2_ to mimic the oxidative stress of cells in a ROS environment and assessed the clearance of ROS by SHC + MXZ using DCFH-DA staining after 24 h. The results were shown in Fig. 4 A and C. A large amount of green fluorescence appeared after H_2_O_2_ stimulation, indicating that the cells were in a state of oxidative stress. After incubation with SHC + MXZ extract, the fluorescence intensity was significantly weakened, suggesting that the oxidative stress of the cells was alleviated, and the normal physiological function was basically restored. Mitochondrial function played an important role for the normal metabolic activities of cells, and under oxidative stress, the cell mitochondrial function was altered, which further led to the exacerbation of inflammation. We assessed the effect of SHC + MXZ on mitochondrial status by staining for mitochondrial membrane potential. At low mitochondrial membrane potential, the JC-1 fluorescent probe accumulates and generates green fluorescence within the mitochondrial matrix, and triggers red fluorescence at high membrane potential [43]. The results were shown in Fig. 4 B, D and E. As could be seen from the figure, H_2_O_2_ stimulation produced a large amount of green fluorescence, indicating that the mitochondrial membrane potential was altered, resulting in impaired mitochondrial function. The incubation of SHC + MXZ microneedle extract prevented the decrease of mitochondrial membrane potential under oxidative stress, and the disappearance of green fluorescence and the production of red fluorescence indicated that the mitochondrial membrane potential was restored. In addition, we measured the changes in ATP content of different groups through actual ATP detection, and the results are shown in Fig. S7 A. The ATP content in the H_2_O_2_ group was significantly reduced, indicating that H_2_O_2_ induced severe mitochondrial energy metabolism dysfunction. However, in the SHC + MXZ group, the ATP content was significantly increased, suggesting that mitochondrial metabolic disorders were alleviated and normal energy supply was restored. We also detected the mRNA expression levels of SOD2 and NOX2 in different groups by qPCR (Fig. S7 B, C). The expression levels of both are highly positively correlated with ROS concentration. Thus, their expression was significantly upregulated in the H_2_O_2_ group. After treatment with SHC + MXZ, their expression was significantly decreased, indicating a substantial reduction in ROS content and inhibition of ROS generation. Therefore, SHC + MXZ microneedles could scavenge ROS and restore mitochondrial function under oxidative stress.

Macrophages were important immune cells that play a key role in wound repair and immune regulation and could be polarized into pro-inflammatory (M1) and anti-inflammatory (M2) types in different environments [44,45]. We induced polarization of RAW 246.7 macrophages towards pro-inflammatory M1 type by lipopolysaccharide (LPS). The expressions of CD86 (M1 type) and CD206 (M2 type) in RAW 246.7 macrophages were assessed using flow cytometry, and the results were shown in Fig. 4. F. The expression of CD86 was significantly increased after LPS induction and decreased after treatment with SHC + MXZ microneedle extract. In contrast, the expression of CD206 was significantly elevated, indicating that macrophages were reversed from M1 type to M2 type. In addition, we quantified CD86/CD206 (Fig. 4 H), and the results showed that SHC + MXZ microneedle extract treatment showed the lowest value. Subsequently, we further verified the macrophage phenotypic switch by immunofluorescence staining, and the results were shown in Fig. 4. G. The marker of pro-inflammatory-type macrophages, iNOS, was significantly increased after LPS induction. The marker of anti-inflammatory type macrophages, CD206, was significantly elevated after incubation with SHC + MXZ microneedle extract, as evidenced by the quantitative statistics of iNOS and CD206 fluorescence intensity (Fig. 4 I, J). The above results suggested that SHC + MXZ microneedles could alleviate the inflammatory environment of macrophages to a certain extent. In summary, this study revealed the multiple beneficial effects of SHC + MXZ microneedles. It not only directly scavenged reactive oxygen species (ROS) but also restored mitochondrial membrane potential that had declined due to oxidative stress, and polarize macrophages from the pro-inflammatory M1 phenotype to the anti-inflammatory M2 phenotype, with inherent synergistic correlations among these effects. On one hand, the well-documented immunomodulatory properties of Zn^2+^ lead to decreased expression of TNF-α by inhibiting the TNF-α signaling pathway. As a major pro-inflammatory cytokine, the reduced level of TNF-α directly alleviated inflammatory damage to mitochondria, facilitating the stabilization of membrane potential and restoration of function. On the other hand, the scavenged ROS and functionally restored mitochondria themselves created an anti-inflammatory microenvironment more conducive to M2 polarization. Therefore, by regulating the TNF-α signaling pathway as a core node, SHC + MXZ simultaneously drived two key repair processes, which was inflammation resolution and mitochondrial function restoration, hereby synergistically accelerating wound healing.

### 2.5 Microneedling for promoting diabetic infected wounds healing

**Fig. 5:**
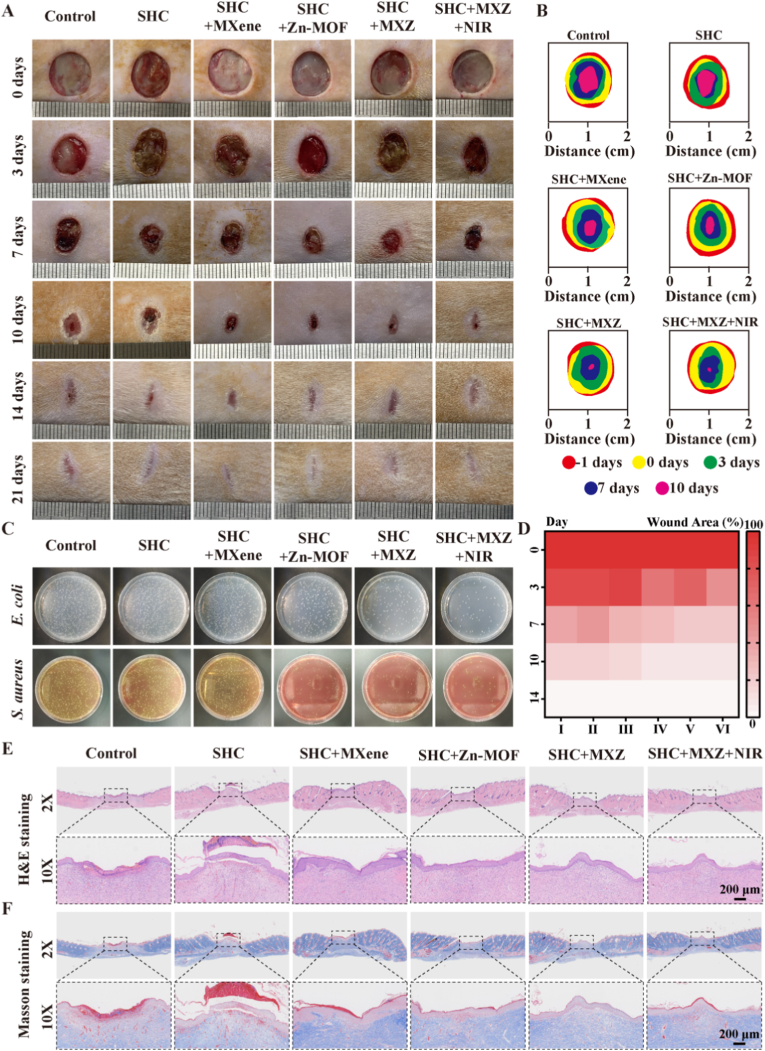
**Microneedling promoted the repair of diabetic infected wounds in rats**. A. Gross view of a diabetic infected wound; B. Tracing diagram of the wound area; C. Number of *E. coli* and *S. aureus* colonies on the wound tissue; D. Heat map of wound area; E. H&E staining of wound tissue; F. Masson staining of wound tissue.

The results *in vitro* demonstrated that SHC + MXZ microneedles had good antimicrobial and immunomodulatory functions and had good potential for application in the field of chronic wound repair. Therefore, we evaluated the effect of SHC + MXZ microneedles on chronic wound repair *in vivo*. Streptozotocin (STZ) was used to induce male SD rats to construct a diabetic model, and after the successful construction of the diabetic model, wounds were created on their backs and bacterial infections were added for 24 h to construct diabetic infected chronic wounds. Subsequently, the wounds were treated using different microneedles, in which the NIR group irradiated the wounds for 10 min using an intensity of power of 1.5 W/cm^2^. The treatment process was shown inFig. S8 A. The wounds were continuously monitored and recorded on days 0, 3, 7, 10, 14, and 21, and the results were shown in Fig. 5 A, B. The macroscopic photographs showed that all wound areas gradually decreased over time, with the SHC + MXZ + NIR treatment group exhibiting the fastest rate of healing. Quantitative statistics on wound area were also carried out and the results were shown in Fig. S8. B and Fig. 5. D. On day 3 the wound area of the SHC + MXZ + NIR group was only 48.5 %, which was significantly lower than the wound area of the control group (83.3 %). Similar results were seen on days 7 and 10, and the above results suggested that SHC + MXZ microneedles could promote the repair of diabetic infected wounds more quickly. To verify the antimicrobial effect of SHC + MXZ microneedles *in vivo*, we took the wound tissue on day 3 and milled it into tissue homogenate and then coated it on selective medium and incubated it in a 37 °C incubator for 24 h, the results were shown in Fig. 5. C. Many colonies existed in the control group, while the number of colonies was significantly reduced after treatment with SHC + MXZ microneedles. In addition, we further quantified the bacterial count, and the results were shown in Fig. S9. Both types of bacteria were present in high numbers in the control group, while their counts were significantly reduced after treatment with SHC + MXZ microneedles, attributed to the antimicrobial activity of Cu ions. Furthermore, after additional NIR irradiation, the bacterial count was further decreased, which was caused by bacterial death induced by photothermal heating. These results indicated that SHC + MXZ microneedles also exhibited excellent antimicrobial ability *in vivo*. To further observe the microscopic repair effect of the wound tissue, we performed H&E staining and Masson staining on the wound tissue, and the results were shown in Fig. 5 E, F and Fig. S10 A, B large number of inflammatory cells accumulated in the control group on day 3, while the inflammatory infiltration of the SHC + MXZ microneedle treatment group species was relatively slight. This was due to the excellent antibacterial effect of SHC + MXZ microneedle to remove bacteria from the wound site in time. After 10 days of treatment, more follicular structures and tighter granulation tissue appeared in the SHC + MXZ microneedling group. In addition, Masson staining results showed more collagen deposition, demonstrating optimal repair. Finally, on day 21, we performed section observations on the heart, liver, spleen, lung, and kidney tissues of the Control group and the SHC + MXZ + NIR group. The results, as shown in Fig. S11, indicated that the cells in both the Control group and the SHC + MXZ + NIR group had clear and normal morphologies, exhibited uniform tissue structures, and no obvious inflammatory response was observed. This verified the excellent *in vivo* biocompatibility of the SHC + MXZ microneedles. Finally, on day 21, we performed section observations on the heart, liver, spleen, lung, and kidney tissues of the control group and the SHC + MXZ + NIR group. The results, as shown in Fig. S11, indicated that the cells in both the control group and the SHC + MXZ + NIR group had clear and normal morphologies, exhibited uniform tissue structures, and no obvious inflammatory response was observed. This verified the excellent *in vivo* biocompatibility of the SHC + MXZ microneedles. Furthermore, we verified the effect of the SHC + MXZ microneedles on hematology through a complete blood count (CBC). As presented in Fig. S12, the results showed that the hematological indices of the SHC + MXZ + NIR group had minimal changes compared with those of the control group, indicating that the hematological parameters were basically stable. The liver and kidney functions after SHC + MXZ + NIR treatment were evaluated via blood biochemistry. The results, shown in Fig. S13, demonstrated that the liver function indices of the SHC + MXZ + NIR group were close to those of the control group, which ruled out the potential risk of liver and kidney damage caused by the metabolic excretion of MXZ. Comprehensively, the above results suggest that SHC + MXZ combined with NIR treatment can significantly promote the repair of diabetic infected wounds without inducing potential cumulative liver toxicity, providing a foundation for further clinical application.

### 2.6 Evaluation of immunofluorescence staining

**Fig. 6:**
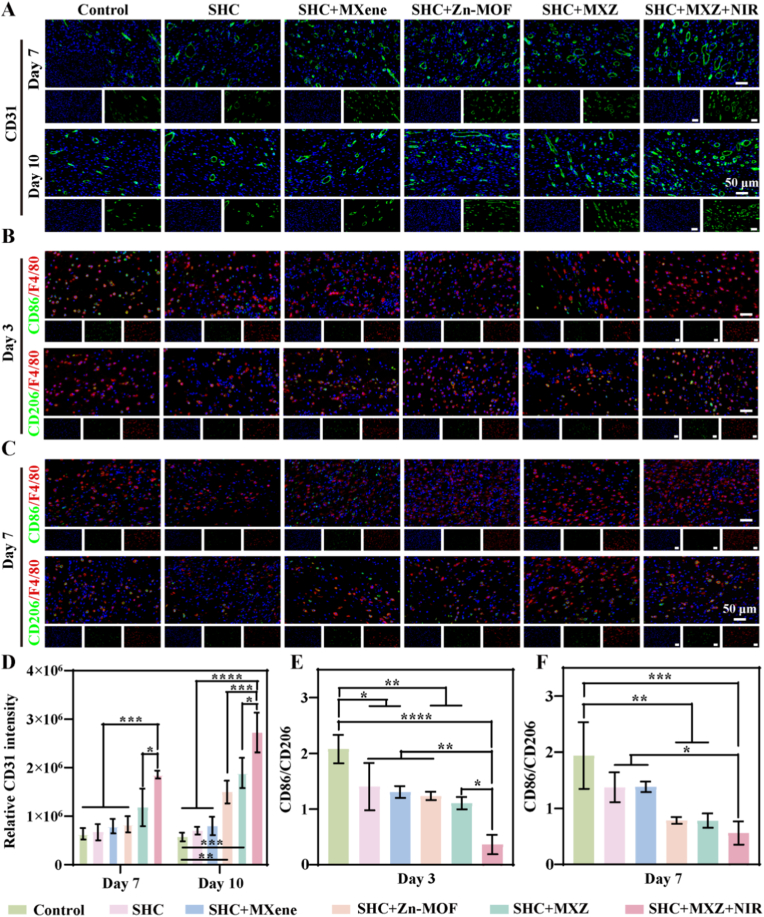
**Immunofluorescence staining of wound tissue**. A. Immunofluorescence staining of CD31; B. Immunofluorescence staining of CD86 and CD206 on day 3; C. Immunofluorescence staining of cd86 and CD206 on day 7; D. Quantitative statistics of CD31 fluorescence intensity; E. CD86/CD206 ratio on day 3; F. CD86/CD206 ratio on day 7.

Angiogenesis was crucial for chronic wound repair, where blood vessels deliver nutrients and oxygen to the granulation tissue in the wound [46]. We had verified *in vitro* that SHC + MXZ microneedles promote angiogenesis through activation of the TGF-β signaling pathway, and further we visually assessed the effect of SHC + MXZ microneedles in promoting angiogenesis *in vivo* by immunofluorescence staining. CD31 was a transmembrane protein expressed during angiogenesis and its expression could be assessed to indicate newly formed vessels [47]. The results were shown in Fig. 6 A, D. At day 7 green fluorescence labelled CD31 was significantly excess in the SHC + MXZ + NIR group over the other groups. In addition, at day 14, SHC + MXZ and SHC + MXZ + NIR showed larger lumen structures. The above results suggested that SHC + MXZ microneedles also had excellent pro-angiogenic ability *in vivo*. Macrophages played an important regulatory role in immune precautionary during chronic wound repair [48]. Early M1-type macrophages release pro-inflammatory factors to exacerbate inflammation, and the repair process gradually shifts to M2-type macrophages releasing anti-inflammatory factors to promote repair. Thus, the gradual shift from M1 to M2 marks the transition from inflammation to the proliferative repair phase. We had verified *in vitro* that SHC + MXZ microneedles could effectively modulate macrophage polarization from M1 to M2 phenotype. Further we had investigated the polarization status of macrophages *in vivo* by labelling M1 macrophages (CD86) and M2 macrophages (CD206) by immunofluorescence staining and quantitatively counting the fluorescence intensity, the results of which were shown in Fig. 6 B, C, E and F. On day 3 the control group showed a strong inflammatory response and after treatment the level of inflammation decreased. On day 7, CD86 in the control group still showed strong green fluorescence, indicating a large infiltration of M1 type macrophages and significant inflammation in the wound tissue. However, fewer M1-type macrophages and more M2-type macrophages were observed in the SHC + MXZ and SHC + MXZ + NIR groups, indicating reduced inflammation. The above results demonstrated that SHC + MXZ microneedles also had an immunomodulatory function *in vivo*.

### 2.7 Immunohistochemistry and western blot evaluation

**Fig. 7:**
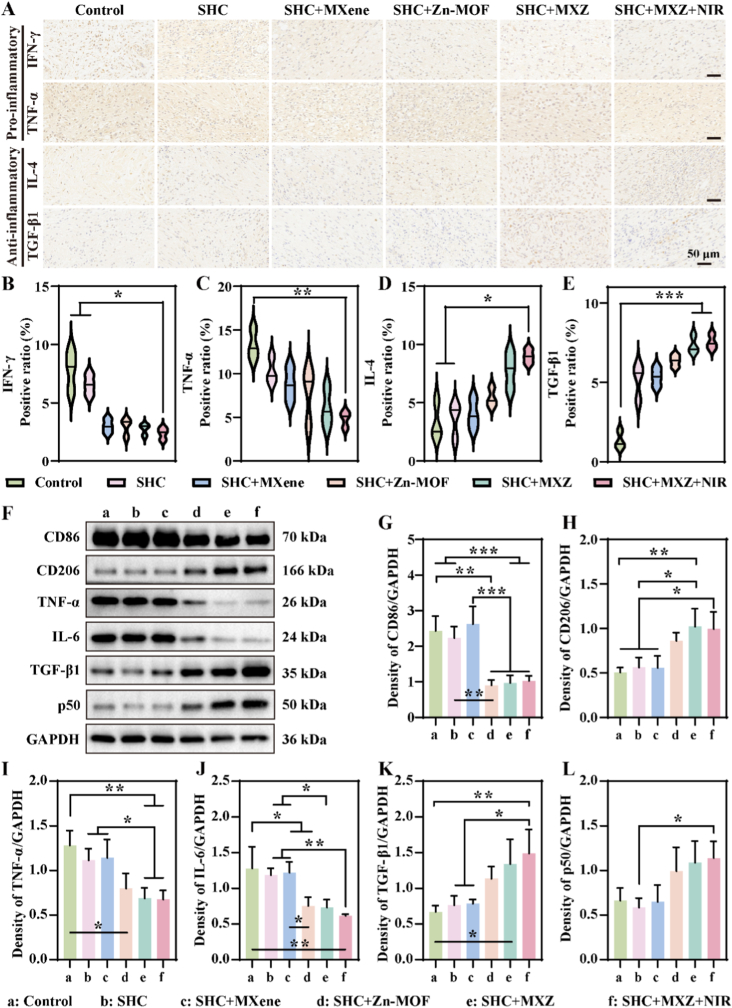
**A. Immunohistochemistry and western blot of wound tissue**. A. Immunohistochemical analysis in wound tissue on day 7; The relative expression level of IFN-γ (B), TNF-α (C), IL-4 (D) and TGF-β1 (E); F. The western blot (WB) bands of the wound tissue; Quantitative statistics of WB bands for CD86 (G), CD206 (H), TNF-α (I), IL-6 (J), TGF-β1 (K) and p50 (L).

We had demonstrated *in vitro* and *in vivo* that SHC + MXZ microneedles could alleviate the inflammatory response at the wound site. It was investigated whether the alleviated inflammatory response and accelerated wound repair correlated with the macrophage polarization status in chronic wounds. Further we assessed the expression levels of pro-inflammatory and anti-inflammatory factors by immunohistochemical staining and the results were shown in Fig. S14 and Fig. 7. A. The results showed that pro-inflammatory factors (IFN-γ and TNF-α) were highly expressed in the control group, and the expression of pro-inflammatory factors was significantly decreased after SHC + MXZ treatment. In contrast, the expression of the anti-inflammatory factors (IL-4 and TGF-β1) were low in the control group and significantly increased after treatment. We also performed quantitative statistics on the expression of inflammatory factors, and the results were shown in Fig. 7 B-E, which had a similar result. Finally, we further verified the expression of proteins associated with inflammation in the trabecular tissue by WB, and the results were shown in Fig. 7. F. Among them, the expressions of CD86 and CD206 were consistent with the previous results of immunofluorescence, further confirming that SHC + MXZ could regulate macrophage polarization towards the M2 phenotype. In addition, the expression of pro-inflammatory factors TNF-α and IL-6 was reduced, and the expression of the anti-inflammatory factor TGF-β1 was increased. We also investigated the mitochondrial functional protein p50 in the wound tissue and showed high expression of p50 in the SHC + MXZ treatment group, demonstrating the restoration of mitochondrial function, which was consistent with the results of mitochondrial membrane potential staining. Finally, we also quantified the grey scale of the WB bands (Fig. 7 G-L), and the results demonstrated that SHC + MXZ microneedles reduced the level of inflammation and restored mitochondrial function, which in turn promoted the repair of chronic wounds.

### 2.8 Transcriptomics sequencing and signaling pathway validation

**Fig. 8:**
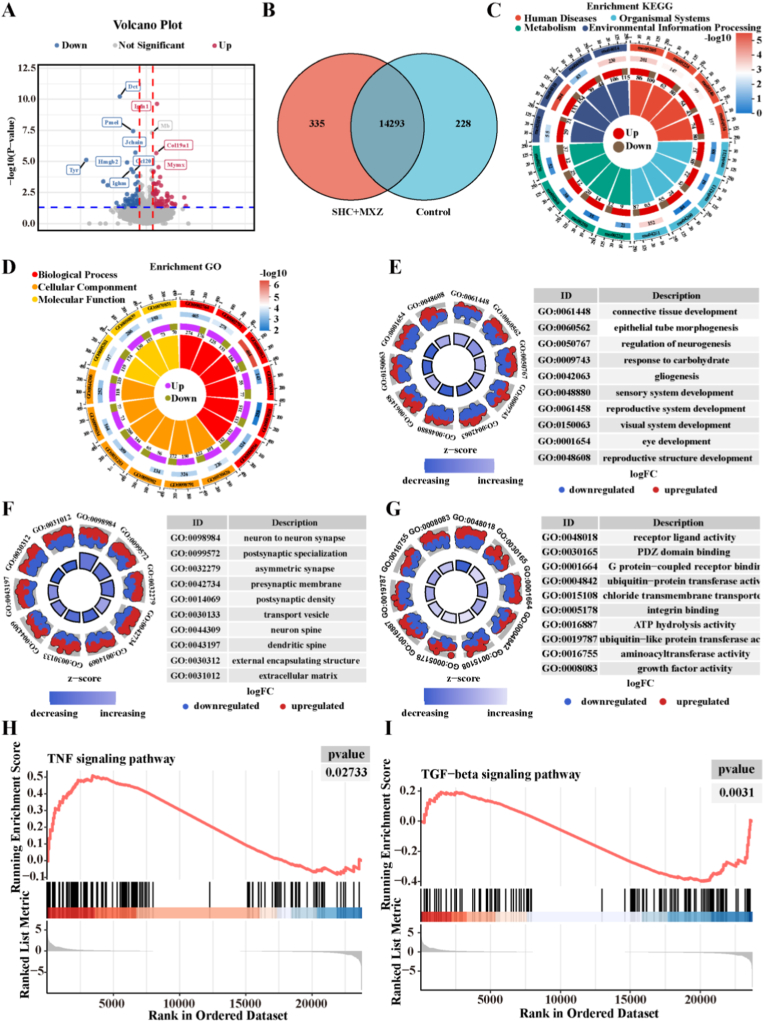
**Transcriptomics sequencing of wound tissue**. A. Volcano plot of differential gene expression (red indicates up-regulated genes, blue indicates down-regulated genes); B. Number of genes expressed in different groups; C. Circle plot of KEGG enrichment; D. Circle plot of GO enrichment; E. GO enrichment of the top 10 of biological processes; F. GO enrichment of the top 10 of cellular component; G. GO enrichment of the top 10 of molecular function; H. GSEA showing TNF signaling pathway; I. GSEA showing TGF-β signaling pathway.

To deeply explore the potential mechanism of SHC + MXZ microneedles to promote chronic wound repair, we selected wound tissues from the control group and the SHC + MXZ group on day 7 for RNA transcriptomic analysis. Fig. S15. A showed the PCA analysis of the control and SHC + MXZ groups, which showed that PC1 (33.98 %) and PC2 (21.80 %) were able to effectively differentiate between the two groups, suggesting that SHC + MXZ microneedling treatment altered gene expression. The expression difference heatmap of gene expression between the two groups was shown in Fig. S15 B. The presence of several genes significantly up-regulated after SHC + MXZ microneedle repair suggested that SHC + MXZ microneedle might promote wound repair by activating the signaling pathways corresponding to certain genes. Subsequently, the up- and down-regulated genes were further analyzed, and the results were shown in Fig. 8 A, B. There existed 14,293 genes co-expressed between the two groups. Among them, 335 genes were specifically expressed in the SHC + MXZ microneedle group, which might promote wound repair by regulating these specific genes. KEGG enrichment analyses (Fig. 8 C) were mainly related to processes associated with human diseases, biological systems, metabolism and environmental information processing. The significant enrichment into environmental information processing suggested that SHC + MXZ microneedles modulate the immune microenvironment of traumatized tissues, which was also an important mechanism for accelerating wound repair. To further clarify the roles played by the differential genes in the repair process, we revealed the effects of SHC + MXZ microbicides on biological processes (BP), cellular functions (CC) and molecular functions (MF) by GO enrichment analysis, and the results were shown in Fig. 8 D-G. The GO enrichment circle plots (Fig. 8 D) indicated the existence of a significant regulation on the three processes of BP, CC and MF. Fig. 8. E further revealed the functions related to BP processes, mainly including connective tissue development, blood vessel formation, etc. CC processes mainly included transport vehicles, extracellular matrixes, etc. (Fig. 8 F). MF processes mainly include ATP hydrolysis activity, growth factor activity, etc. (Fig. 8 G). To further analyze the effect of SHC + MXZ microneedles on signaling pathways, functional enrichment analysis was performed using GSEA. The TNF signaling pathway (Fig. 8 H) was mainly involved in inflammatory regulation and immune response, which had been confirmed by the expression of inflammatory factors. The TGF-β signaling pathway (Fig. 8 I) mainly promoted angiogenesis to accelerate wound repair, and this signaling pathway had also been validated *in vitro* and *in vivo*. Finally, protein-protein interaction network analysis (PPI) also revealed that SHC + MXZ microneedles modulated the TNF signaling pathway (Fig. S15 C) and TGF-β signaling pathway (Fig. S15 D). In conclusion, SHC + MXZ microneedles could accelerate chronic wound repair through various mechanisms such as immunomodulation, anti-inflammation, regulation of mitochondrial function and promotion of angiogenesis.

## 3 Conclusion

In summary, we designed a novel bilayer SCS/HAMA/Col_III_MA (SHC) multifunctional microneedle loaded with MXene@Zn-MOF (MXZ) composites for the repair of diabetic infected wounds. The MXZ composites had excellent photothermal and antimicrobial properties, which were capable of effectively killing bacteria and removing bacterial biofilms. In addition, SHC + MXZ microneedles had excellent biocompatibility and promoted angiogenesis by activating the TGF-β signaling pathway. It could also inhibit the TNF-α signaling pathway to reduce inflammation and thus modulate the local immune microenvironment of diabetic infected wounds. The results of the mitochondrial membrane potential study showed that SHC + MXZ microneedles could also effectively reduce the ROS level of L929 cells and regulate the mitochondrial function, thus restoring the energy supply of diabetic infected wounds. Further treatment with SHC + MXZ microneedles in diabetic wounds infected with *E. coli* and *S. aureus* effectively cleared the bacteria and showed good pro-vascularization and immunomodulation effects, significantly promoting the repair of diabetic infected wounds. Finally, the gene expression after repair by SHC + MXZ microneedles was verified by transcriptomic sequencing of the wound tissue, and the relevance of TGF-β and TNF-α signaling pathways was verified, which elucidated the intrinsic mechanism of SHC + MXZ microneedling for repairing diabetic chronic wounds.

Although our study has demonstrated the potential therapeutic value of SHC + MXZ microneedles in diabetic wound repair, several limitations should be acknowledged. We confirmed the involvement of the TGF-β and TNF-α signaling pathways through transcriptomic analysis and inhibitor experiments, but more detailed molecular mechanisms—such as specific upstream regulatory factors and downstream effector genes—remain to be further investigated and elucidated. Additionally, this study did not fully examine the biodegradation kinetics and metabolic pathways of the MXene and Zn-MOF components in the composite in *vivo*. The in *vivo* validation was conducted in an acute diabetic infected wound model, and the efficacy of this system in more complex chronic clinical scenarios using large animal models requires further exploration.

## 4 Experimental section

*Materials*: Ti_3_AlC_2_ MXene was purchased from Forsman scientific (Beijing) Co., Ltd. Lithium fluoride (LiF) was purchased from Meryer (Shanghai) Chemical Technology Co., Ltd. Hydrochloric acid (HCl) and N,N-dimethylformamide (DMF) was from Guangzhou Chemical Reagent Factory. Zinc acetate, 2-methylimidazole, methanol, chitosan, and methacrylic anhydride were purchased from Macklin Biotechnology Co. Hyaluronic acid was purchased from Shanghai Yuanye Biotechnology Co. Collagen III was obtained from Jiangsu JLand Biotech Co., Ltd. (Jiangsu, China). HClSO_3_ was purchased from J&K Scientific.

*Synthesis of MXZ*: Ti_3_AlC_2_ (250.0 mg) and LiF powder (400.0 mg) were dissolved in HCl solution (5.0 mL, 9 mol/L) and etching was carried out by stirring for 24 h at 40 °C, and at the end of the etching, the multilayered Ti_3_C_2_ product was cleaned up to the pH of the supernatant reached 7.0. The etched multilayer Ti_3_C_2_ nanosheets were peeled off by ultrasound (200 W, 30 min), followed by several centrifugation (3500 rpm, 5 min) and mixing operations to obtain a monolayer of Ti_3_C_2_ MXene by taking the upper liquid layer. 8.87 g of 2-methylimidazole was weighed and dissolved in 200 mL of deionized water, then 500 mg of zinc acetate-dihydrate was added and stirred at room temperature for 24 h. The supernatant was discarded by centrifugation at 8000 rpm for 20 min to obtain the Zn-MOF. The monolayer of Ti_3_C_2_ MXene (10 mg) and Zn-MOF (10 mg) were dispersed in 10 mL of deionized water, sonicated for 10 min to obtain a mixed solution, and freeze-dried at −80 °C to obtain the MXZ.

*Preparation of HAMA and Col*_*III*_*MA*: 5.0 g of hyaluronic acid was dissolved in 400 mL of deionized water, 12 mL of methacrylic anhydride was added to the solution, and the pH of the reaction solution was adjusted to 8.5 with 5 M NaOH. The reaction solution was stirred for 24 h at room temperature, and HAMA was obtained by dialysis followed by freeze-drying. 10 g of Col_III_ was dissolved in 500 mL of water, add 10 mL of methacrylic anhydride to the solution and stir at room temperature for 24 h. After dialysis and centrifugation (10,000 rpm, 15 min) to remove the impurities, the supernatant was freeze-dried to obtain Col_III_MA.

*Preparation of SCS*: 50 mL of DMF solution was added into a 100 mL beaker and cooled down in an ice bath at 0–4 °C, then 5 mL of HClSO_3_ was added drop by drop into the cooled DMF solution, stirred and cooled to room temperature to obtain the sulfonation reagent. 1.5 g of chitosan was added to 50 mL of formamide and 2 mL of formic acid and mechanically stirred to dissolve chitosan completely. Under the condition of N_2_ protection, the sulfonation reagent was slowly dripped into the mixture, and the reaction was carried out for 2 h at 50 °C. After the reaction was completed, 500 mL of anhydrous ethanol was added to the reaction solution to precipitate the product, which was filtered and washed. The washed solid was dissolved in 100 mL of deionized water and the pH was adjusted to 7–8. The solution was centrifuged (8000 rpm, 10 min) and the supernatant was taken, dialyzed and freeze-dried to obtain SCS.

*Preparation of SHC microneedles*: a mixture of 2 % (w/v) HAMA and 1 % (w/v) SCS was added to the PDMS mould, and the mould was placed in a vacuum chamber to remove all air bubbles to ensure that the tip was filled completely. After filling, the tip layer was cross-linked using blue light irradiation for 30 s to form the tip layer. 10 % (w/v) of Col_III_MA was added to the PDMS mould that had been filled with the tip, the vacuum was repeated in a vacuum chamber to expel the gas, and blue light irradiation for 30 s to cross-link to form the substrate layer. Finally, the SCS/HAMA/Col_III_MA (SHC) microneedles were removed from the mould after natural air drying and stored in a desiccator. 1 mg of MXene, 1 mg of Zn-MOF and 1 mg of MXZ were dispersed in 1 mL of 10 % Col_III_MA solution, respectively, and the Col_III_MA solution containing MXene, Zn-MOF and MXZ was subsequently added to the PDMS mould. SHC + MXene, SHC + Zn-MOF and SHC + MXZ microneedles were obtained after drying and demoulding.

*FT-IR*: 3–5 mg of CS and SCS were taken with appropriate amount of dried potassium bromide powder and ground thoroughly and pressed to obtain thin samples. The scanning range was set to 4000-400 cm^−1^ and detected using FT-IR spectrometer.

*H*^*1*^ *NMR*: Col_III_, Col_III_MA, HA and HAMA were fully dissolved in deuterated heavy water as a solvent and dissolved into clean NMR tubes. The H^1^ NMR structure was determined using H^1^ NMR spectroscopy at room temperature.

*SEM*: Multilayer MXene, singlelayer MXene, Zn-MOF, MXZ and SHC microneedles were glued on a sample stage with conductive adhesive and gold was sprayed on the surface of the samples for 30 s. The surface morphology of the microneedles was observed using a scanning electron microscope, and the elements were detected at different locations on the sample surface by EDS.

*XRD*: Zn-MOF, MXene and MXZ were loaded flat to fill the sample stage and keep the test surface flat and smooth, the sample stage was fixed to the instrument to test the structure of the materials using X-ray polycrystalline powder diffractometer (XRD).

*Microneedle puncture and mechanical strength*: SHC microneedles were pressed against the pigskin and the formed needle holes were photographed using a mobile phone macro camera. For puncture depth, SHC microneedles were used to zap agarose, and fluorescence images were acquired using a laser confocal fluorescence microscope at 20 μm scanning intervals along the Z-axis to observe the depth of SHC microneedle puncture after puncture. For mechanical strength testing, the tip of the SHC microneedle was turned upwards and the tip was compressed downwards using a universal stress tester. The displacement and force generated during compression were recorded to assess the mechanical strength of the microneedle.

*Zeta potential*: Zn-MOF, MXene and MXZ were ultrasonically dispersed in deionized water, and the solution was subsequently transferred into a cuvette and placed in a dynamic light scatterometer. The potentials of Zn-MOF, MXene and MXZ were detected by a dynamic light scatterometer.

*Photothermal properties*: The MXZ solution and SHC + MXZ microneedles were irradiated and warmed up using an 808 nm near-infrared laser, the real-time temperature of the MXZ solution was recorded using a thermocouple thermometer, and an infrared thermography camera was used to obtain the real-time thermal images of the SHC + MXZ microneedles. Different concentrations (0, 0.25, 0.5, 0.75, 1.0 mg/mL) of MXZ solution were configured, and the laser power was adjusted (0.5, 1.0, 1.5, 2.0 W/cm^2^) to irradiate the MXZ solution, and real-time temperature of the MXZ was recorded every 20 s. The temperature rise curve was plotted with time as the horizontal coordinate and temperature as the vertical coordinate. For cyclic photothermal, an 808 nm NIR laser was used to irradiate the MXZ solution for 10 min and then stop irradiation to reduce the temperature of the MXZ solution to the initial temperature. This operation was repeated 5 times, and the real-time temperature of the MXZ solution was recorded every 20 s to plot the temperature increase-cooling cycle curve. The photothermal efficiency was calculated according to the following equation:

*Bacterial plate count:* Inoculate *E. coli* and *S. aureus* bacterial solution onto LB agar plate and incubate for 24 h, select single colonies to be inoculated in LB liquid medium and incubate at 37 °C on a constant temperature shaker for 4 h for the subsequent experiments. Corresponding microneedles were added to the 24-well plates, followed by 100 μL of 1 × 10^8^ CFU/mL of bacterial solution on the microneedles, and incubated for 24 h after adding 1 mL of sterile saline. After the culture was completed, the bacterial solution was diluted and coated on LB agar plates and incubated for 24 h. The number of colonies was counted, and the bacterial inhibition rate was calculated.

*Bacterial live-dead staining*: Bacteria co-cultured with microneedles were centrifuged to remove the culture medium, resuspended with 1 mL of PBS and added with a certain amount of dead/live bacterial fluorescent staining reagent (Syto-9: PI = 1:1), and stained by avoiding light for 15 min. After centrifugation, the excess dye was removed by washing with PBS, and the pictures were taken for observation by fluorescence microscope.

*Bacterial SEM*: Bacteria co-cultured with microneedles were centrifuged to remove the culture solution, and the bacterial precipitate obtained by centrifugation was immobilized with 1 mL of 2.5 % glutaraldehyde. After centrifugal cleaning and resuspension in deionized water, the resuspended bacterial liquid was taken and dropped on a single crystal silicon wafer. After drying naturally, it was stuck on the sample stage with conductive adhesive and observed after spraying gold.

*Biofilm crystal violet staining*: 200 μL of bacterial suspension was added to the 48-well plate with the corresponding microneedle, followed by 500 μL of LB liquid medium. The plates were incubated at 37 °C for 72 h, and the medium was replaced with fresh LB medium every 12 h. After incubation, 500 μL of anhydrous methanol was used for the incubation. After incubation, the biofilm was fixed with 500 μL of anhydrous methanol, washed, and then stained with 200 μL of 1 % crystal violet staining solution, avoiding light for 30 min. After washing and drying at room temperature, 500 μL of anhydrous ethanol was added to each well until all the crystal violet was dissolved, and the absorbance value at 570 nm was photographed and measured.

*Biofilm Live-Dead Staining*: Add 200 μL of bacterial suspension to a confocal petri dish with the corresponding microneedle, followed by 1 mL of LB liquid medium. Incubate at 37 °C for 72 h and replace the medium with fresh LB liquid medium every 12 h. After incubation, live/dead bacteria fluorescent staining reagent (Syto-9: PI = 1:1) was added, and the staining was protected from light for 30 min. Excess dye was removed by washing with PBS and then observed and photographed by laser confocal fluorescence microscope.

*MXZ concentration screening*: MXZ solutions with concentrations of 0.1, 0.25, 0.5, 1 and 2.5 mg/mL were prepared in DMEM high-glucose medium and set aside. L929 cells were digested and resuspended and then inoculated into 96-well plates with 5000 cells/well, and cultured for 24 h to wait for the cells to attach to the wall. The cells were incubated for 24 h with different concentrations of the MXZ culture solution, then the liquid in the wells was aspirated, and each well was incubated with 100 μL of CCK-8 working solution for 30 min. The absorbance (OD) was measured at 450 nm with an enzyme marker, and the cell viability was calculated according to the following equation:

$$\text{cell}\;\text{viability}\left(\%\right)=\frac{{\text{OD}}_{\text{Experimental}}-{\text{OD}}_{\text{Blank}}}{{\text{OD}}_{\text{Control}}-{\text{OD}}_{\text{Blank}}}\times 100\%$$

*Microneedle toxicity test*: The biocompatible concentrations were screened according to the MXZ concentration, and then the corresponding grouped microneedles were formulated according to the selected concentrations (SHC, SHC + MXene, SHC + Zn-MOF, SHC + MXZ). Microneedle extracts were obtained by submerging with DMEM high-glucose medium at a ratio of 0.1 g/m L for 24 h at 37 °C. L929 cells were digested and resuspended and then inoculated into 48-well plates at a cell number of 10,000 cells/well. The cells were cultured for 24 h, and then added with different microneedle extracts and continued to be cultured for 24, 48 and 72 h. After the incubation to the corresponding time point, each well was incubated with 300 μL of CCK-8 working solution for 30 min. The absorbance (OD) was measured at 450 nm with an enzyme marker, and the cell viability was calculated according to the following equation:

$$\text{cell}\;\text{viabilit}y\left(\%\right)=\frac{{\text{OD}}_{\text{Experimental}}-{\text{OD}}_{\text{Blank}}}{{\text{OD}}_{\text{Control}}-{\text{OD}}_{\text{Blank}}}\times 100\%$$

*Cell live-dead staining*: The biocompatible concentration was screened according to the MXZ concentration, and the corresponding grouped microneedles (SHC, SHC + MXene, SHC + Zn-MOF, SHC + MXZ) were subsequently formulated according to the selected concentration. Microneedle extracts were obtained by submerging with DMEM high-glucose medium at a ratio of 0.1 g/m L for 24 h at 37 °C. L929 cells were digested and resuspended and then inoculated into 48-well plates at a cell number of 10,000 cells/well. The cells were cultured for 24 h until the cells were attached to the wall, and then different microneedle extracts were added to continue the culture for 24, 48 and 72 h. The cells were cultured until the corresponding time points. The medium in the wells was aspirated, washed with PBS and incubated for 20 min by adding live-dead staining working solution, and the pictures were taken by fluorescence microscope after adding anti-fluorescence quencher to analyze the cell live-dead.

*Cytoskeleton staining*: the steps of culture were the same as cell live-dead staining, after the culture was completed and washed, ghost pen cyclic peptide-labelled rhodamine B staining was added, and the images were taken by fluorescence microscope after incubation.

*Cell Scratching*: Digested L929 cells were resuspended in DMEM high-glucose medium inoculated in 24-well plates and cultured for 24 h to form a confluent monolayer. A straight line was drawn down the cell layer with a 20 μL pipette tip, washed with PBS and cell debris removed. The culture continued by adding different microneedle extracts, and the control group was added with an equal amount of DMEM high-glucose medium. The cell scratch images were taken at 0, 6 and 24 h and the scratch area was counted.

*In vitro tube formation*: Thawed Matrigel was spread onto 48-well plates at 100 μL/well and incubated for 30 min to convert it from liquid to solid gel. HUVECs cells were dispersed by different groups of microneedle extracts and then inoculated onto the surface of Matrigel. The tubular mesh structure formed by the cells was observed by microscope after 6 h of incubation at 37 °C, and the number of nodes, the number of meshes and the number of backbones were counted.

*Flow cytometry*: RAW264.7 was inoculated into 24-well plates, cultured overnight and then induced with 5 μg/mL lipopolysaccharide (LPS) overnight. Replace the microneedle extracts of different groups to continue the culture for 24 h. LPS and PBS were used as the positive control group and negative control group, respectively. After overnight incubation, the cell suspension was digested and collected and incubated with CD86 and CD206 fluorescent antibodies at room temperature for 1 h. The percentages of M1 and M2 were calculated by flow cytometry after the incubation was completed.

*Immunofluorescence staining*: RAW264.7 cells were inoculated into 6-well plates that had been lined with cell crawlers, cultured overnight and then induced with 5 μg/mL of lipopolysaccharide (LPS) overnight. The culture was continued by replacing different groups of microneedle extracts for 24 h. After washing, 1 % paraformaldehyde was added for 15 min for fixation, membrane-breaking solution was added for treatment and washing, and 1 % BSA sealing solution was added for closure. Subsequently, a fluorescent antibody dilution containing rabbit anti-iNOS and mouse anti-CD206 (dilution ratio 1:200) was added and incubated at room temperature. After incubation, FITC Goat Anti-rabbit IgG and Cy3 Goat Anti-mouse IgG fluorescent secondary antibodies were added and incubated at room temperature. After incubation, the slices were blocked with DAPI-containing anti-fluorescence quencher and images were captured in a fluorescence microscope.

*Cellular ROS clearance*: L929 cells were digested and resuspended and inoculated in 48-well plates, cultured for 24 h to wait for cell attachment. Oxidative stimulation was performed by adding H_2_O_2_ (100 μM) for 30 min, followed by aspiration of the liquid. PBS was used to wash and added with different groupings of microneedle extracts to continue the incubation for 24 h. After washing, 10 μM DCFH-DA working solution was added for incubation and then added to DMEM high-glucose medium to maintain the cellular activity and images were taken using fluorescence microscopy.

*Mitochondrial membrane potential staining*: L929 cells were digested and resuspended and inoculated into 6-well plates, cultured for 24 h to allow the cells to attach to the wall, and then added with different groups of microneedle extracts and continued to be cultured for 24 h. Mitochondrial damage was carried out by adding 1:1000 JC-1 working solution, and then added with DMEM high-glucose medium after the incubation, to maintain the activity of the cells and to capture the images with a fluorescence microscope.

*Western blot*: HUVESs cells were digested and resuspended and then inoculated in 6-well plates, cultured for 24 h to wait for the cells to attach to the wall, and then added with different groups of microneedle extracts to continue to be cultured for 24h. The well plates were placed on ice, the liquid in the wells was aspirated, washed 3 times with PBS, RIPA protein lysate containing phosphatase protein inhibitor was added, milled, and placed on ice for 1h to be lysed. Protein samples were obtained by centrifugation of the collected liquid, and the protein was denatured at high temperature. The samples were loaded on gel electrophoresis and undergo electrophoresis, membrane transfer, antibody incubation and development. Protein bands were developed in the developer and collected and analyzed.

*PCR*: HUVECs cells were digested and resuspended and then inoculated in 6-well plates, cultured for 24 h until the cells were attached to the wall, and then added with different groups of microneedle extracts and continued to be cultured for 24 h. The well plate was placed on ice, the liquid in the wells was aspirated and washed 3 times with PBS. RNA extraction solution was added to lysis, and the lysate was collected and extracted to obtain RNA. RNA was added to the gDNA adsorption column after detection of concentration, and the DNA-removed RNA was collected by centrifugation. cDNA was denatured at 65 °C, cooled on ice and subjected to a reverse transcription reaction. Set up the PCR instrument reaction procedure, the cDNA and the reaction solution will be compounded and tested on the machine to get the melting curve and amplification curve, and the data will be exported and analyzed.

*Animal modelling*: A rat diabetes model was constructed by intraperitoneal injection of STZ (65 mg/kg), and fasting blood glucose was monitored for 3 consecutive days, and the modelling was considered successful when the blood glucose concentration was higher than 16 mmol/L for 3 consecutive days. The rats were then anaesthetized by intraperitoneal injection of 0.5 mL of 1 % pentobarbital sodium solution, and four wounds of 10 mm in diameter were created using surgical scissors after removing hair from the back. A 50 μL 1 x 10^8^ CFU/mL mixture of *E. coli* and *S. aureus* was added to each wound drop, and simple debridement using sterile saline was performed 24 h after infection. All the rats that were successfully modeled were randomly divided into 6 experimental groups of 5 in each group, which was using a random number table, with stratification based on body weight and baseline blood glucose levels to ensure inter-group comparability. The treatment was carried out using corresponding microneedles to puncture the wound site, respectively. The control group was directly exposed to the external environment, and the NIR treatment group was irradiated with a laser with a power of 1.5 W/cm^2^ for 10 min. On days 0, 3, 7, 10, 14 and 21 of treatment, the wound area was recorded using a digital camera and measured using image J software. The wound healing rate was calculated using the following equation:

$$\text{Wound}\;\text{healing}\;\text{rate}\;\left(\%\right)=\frac{S0-\text{St}}{S0}\times 100\%$$

Where, St was the wound area at different time points, S0 was the initial wound area.

*In vivo antimicrobial effect*: Day 3 wound tissue was removed and placed in sterilized 1 mL of saline and the wound tissue was ground to obtain a tissue homogenate. Subsequently, the tissue homogenate was diluted with sterile saline and coated with Gram-negative medium and Gram-positive plates. The plates were inverted and incubated at 37 °C for 24 h. Photographs were taken to record the plates and count the number of colonies.

*H&E staining*: Fresh tissues were removed and fixed in 4 % paraformaldehyde, the fixed tissues were dehydrated and embedded in paraffin. Sections were made at a thickness of 4 μm using a paraffin slicer, then spread on slides and deparaffinized. After washing, Harris Hematoxylin Stain and Eosin Stain were added sequentially, and the sections were dehydrated again and sealed with neutral gum. Finally, images were captured and analyzed under a microscope.

*Masson staining*: Paraffin sections were spread on slides and deparaffinized, washed and then sequentially stained with Weigert's iron hematoxylin stain and Reichhorn red acidic magenta stain. Subsequently, the sections were treated with aqueous phosphomolybdic acid and restained with aniline blue solution. After final dehydration, the slices were sealed with neutral gum and images were captured and analyzed under a microscope.

*Immunofluorescence staining*: Paraffin sections were deparaffinized and antigenically repaired using EDTA antigen repair buffer, after which the tissues were sealed with 3 % BSA. After removing the sealing solution, the sections were incubated overnight with a drop of primary antibody, washed, and then covered with a secondary antibody of the corresponding primary antibody species and incubated. Subsequently, the sections were incubated with a drop of DAPI stain and then sealed with an anti-fluorescence quenching sealer. Finally, images were acquired and analyzed under an inverted fluorescence microscope.

*Immunohistochemistry*: Paraffin sections were deparaffinized and antigenically repaired using EDTA antigen repair buffer. After repair, endogenous peroxidase was blocked with hydrogen peroxide, and the tissue was sealed with 3 % BSA. After removal of the sealing solution, the sections were incubated overnight with a drop of primary antibody, washed, and a secondary antibody corresponding to the primary antibody species was added to cover the tissue and incubated. Freshly prepared DAB chromogenic solution was then added, and the nuclei were stained with Harris hematoxylin. Finally, the sections were dehydrated and sealed with neutral gum, and images were captured and analyzed under a microscope.

*Western blot*: Remove the animal tissue in a centrifuge tube and put it on ice, add RIPA protein lysis solution, grind it and put it on ice for 1 h. Collect the liquid and centrifuge it to get the protein samples, and denature the protein at high temperature. The samples were then subjected to gel electrophoresis, followed by electrophoresis, membrane transfer, antibody incubation and development. Protein bands were developed in a developer and collected and analyzed.

*Bioinformatics analysis*: We selected wound tissues from the control group and the SHC + MXZ group on day 7 for transcriptome sequencing. Differentially expressed genes (DEGs) were obtained by Log_2_ Fold Change ≥1, P < 0.05. GO enrichment analysis was obtained by importing DEGs into the Database for Annotation, Visualisation and Integrated Discovery (DAVID) database. Biological processes, cellular component or molecular function locations enriched in DEGs were identified. KEGG enrichment analysis identifies the roles of DEGs in various biological pathways, providing a comprehensive annotation of gene and gene product functions. Their involvement in pathways such as cell signaling and metabolic processes is also analyzed. The collection of genes identified as significantly enriched was subsequently downloaded in the GSEA database and GSEA analysis was performed using R software to enable visual screening to with TNF and TGF-β signaling pathways.

*Statistical Analysis:* All statistical data in this study were expressed as mean ± standard error (n > 3), and data analysis was performed using GraphPad Prism software and Origin software. One-Way Analysis of Variance (ANOVA) was employed to assess the statistical differences among groups. A p-value of less than 0.05 was considered statistically significant (∗p < 0.05, ∗∗p < 0.01, ∗∗∗p < 0.001).

## CRediT authorship contribution statement

**Minjian Liao:** Writing – original draft, Investigation. **Xinmin Guo:** Investigation, Data curation. **Longbao Feng:** Investigation. **Qing Peng:** Investigation, Conceptualization. **Jianhao Liang:** Investigation. **Aleh Kuzniatsou:** Investigation. **Rui Guo:** Writing – review & editing, Supervision, Funding acquisition. **Pan Yu:** Supervision, Funding acquisition. **Shuqin Zhou:** Writing – review & editing, Supervision.

## Animal ethics affirmation

This study contains animal subjects, and the animal experiments were approved by Institutional Animal Care and Use Committee (IACUC) of Ruige Biotechnology. The endorsement number is No. 20240905-001.

## Declaration of competing interest

The authors declare no competing financial interest.

## Data Availability

The data that has been used is confidential.
