# Supplementary material for: Immunomodulatory microneedles restore mitochondrial homeostasis and balance TGF-β/TNF-α signaling to accelerate diabetic wound repair
Source: Mater Today Bio. 2025 Dec 14;36:102687. doi: 10.1016/j.mtbio.2025.102687 (PMC12774784; doi:10.1016/j.mtbio.2025.102687)
Supplement: Multimedia component 1 [file mmc1.docx]

**Immunomodulatory Microneedles Restore Mitochondrial Homeostasis and Balance TGF-β/TNF-α Signaling to Accelerate Diabetic Wound Repair**

Minjian Liao ^a, 1^, Xinmin Guo ^b, 1^, Longbao Feng ^a^, Qing Peng ^c^, Jianhao Liang ^a^, Aleh Kuzniatsou ^d^, Rui Guo ^a, *^, Pan Yu ^e, *^, Shuqin Zhou ^f, g, *^

^a^ Key Laboratory of Biomaterials of Guangdong Higher Education Institutes, Key Laboratory of Regenerative Medicine of Ministry of Education, Guangdong Provincial Engineering and Technological Research Centre for Drug Carrier Development, Department of Biomedical Engineering, Jinan University, Guangzhou, 510632, China.

^b.^ Department of Ultrasound, Guangzhou Red Cross Hospital of Jinan University, Guangzhou, 510220, China.

^c^ Central Laboratory of The Second Affiliated Hospital, School of Medicine, The Chinese University of Hong Kong, Shenzhen & Longgang District People’s Hospital of Shenzhen, Shenzhen, 518172, China.

^d^ Institute of Biochemistry of Biologically Active Compounds of the National Academy of Sciences of Belarus, Grodno, Belarus.

^e^ Department of Burn and Plastic Surgery, Jinling Hospital, Affiliated Hospital of Medical School, Nanjing University, Nanjing, China.

^f^ Department of Anesthesiology of The Second Affiliated Hospital, School of Medicine, The Chinese University of Hong Kong, Shenzhen & Longgang District People’s Hospital of Shenzhen, Shenzhen, 518172, China.

^g^ Department of Anesthesiology, Zhujiang Hospital, Southern Medical University, Guangzhou, 510000, China.

^1^ These authors contributed equally to this work.

* Corresponding author: guorui@jnu.edu.cn (Rui Guo), yp52@163.com (Pan Yu), zhoushuqin@smu.edu.cn (Shuqin Zhou).


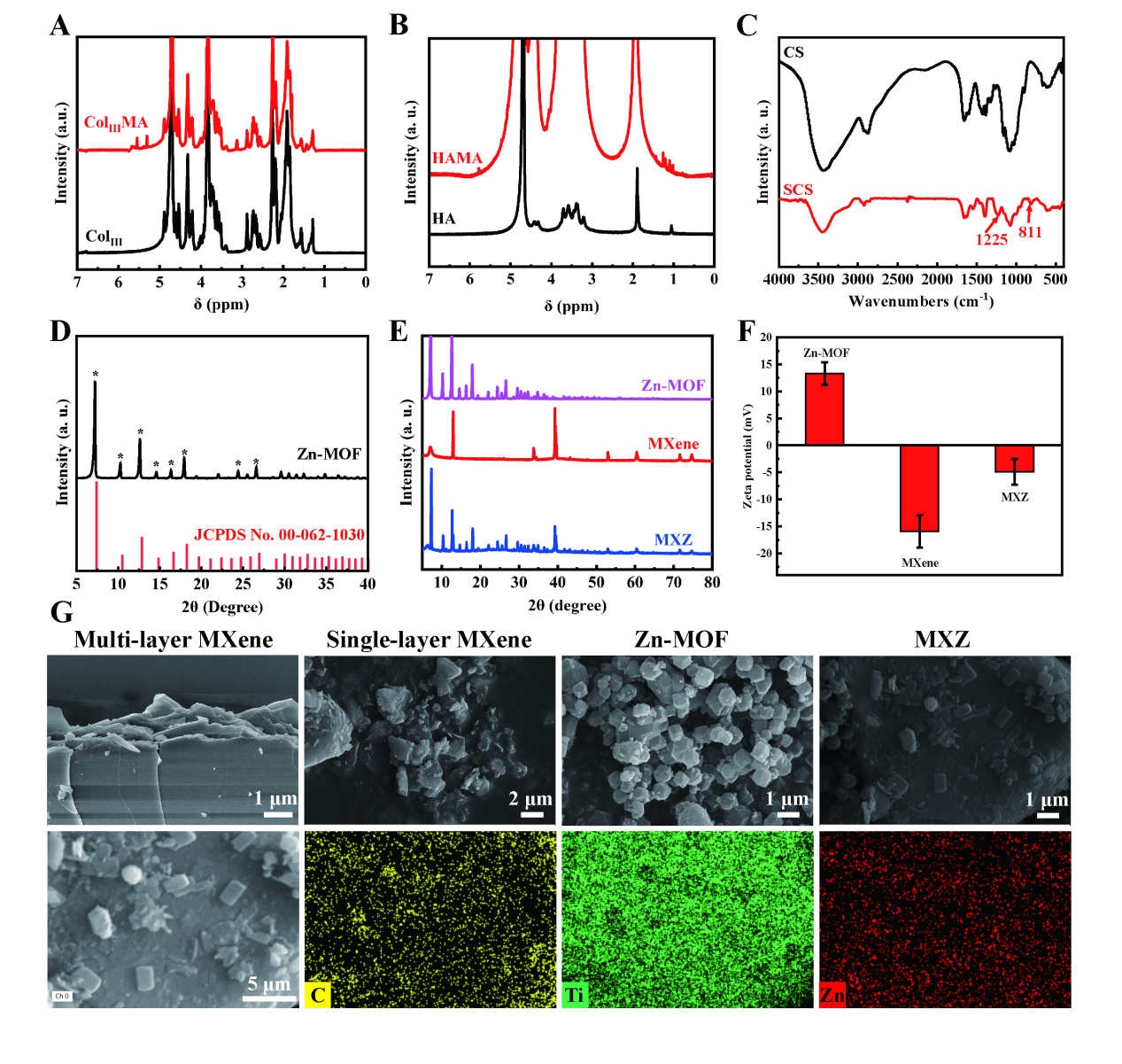


**Fig. S1.** A. ^1^H-NMR image of Col modification; B. ^1^H-NMR image of HA modification; C. FT-IR image of CS modification; D. XRD diagram of Zn-MOF; E. XRD diagram of MXene, Zn-MOF and MXZ; F. Zeta potential diagram of MXene, Zn-MOF and MXZ; G. SEM diagram and elemental distributions of multi-layer MXene, single-layer MXene, Zn-MOF and MXZ.


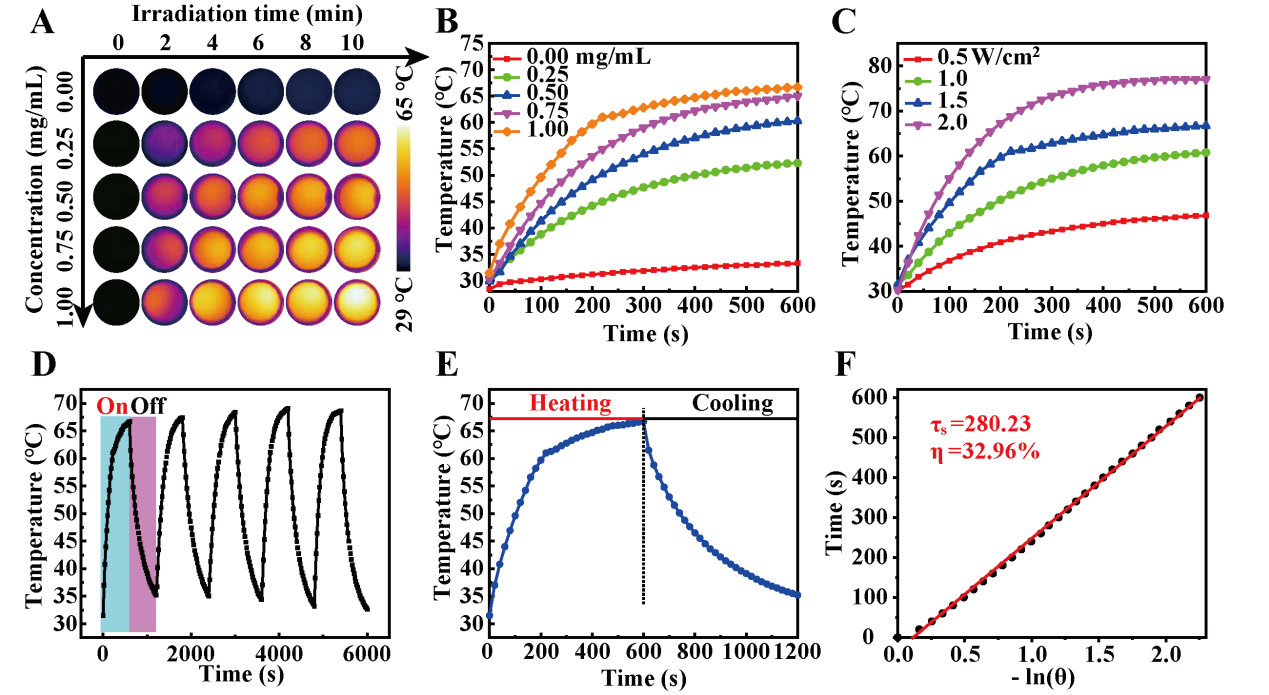


**Fig. S2.** A. Thermogram of a microneedle; B. Photothermal warming curves for different concentrations of MXZ; C. Photothermal warming curves for different powers; D. Photothermal cycling curves; E. Photothermal warming and cooling curves; F. Photothermal time constant and photothermal efficiency.


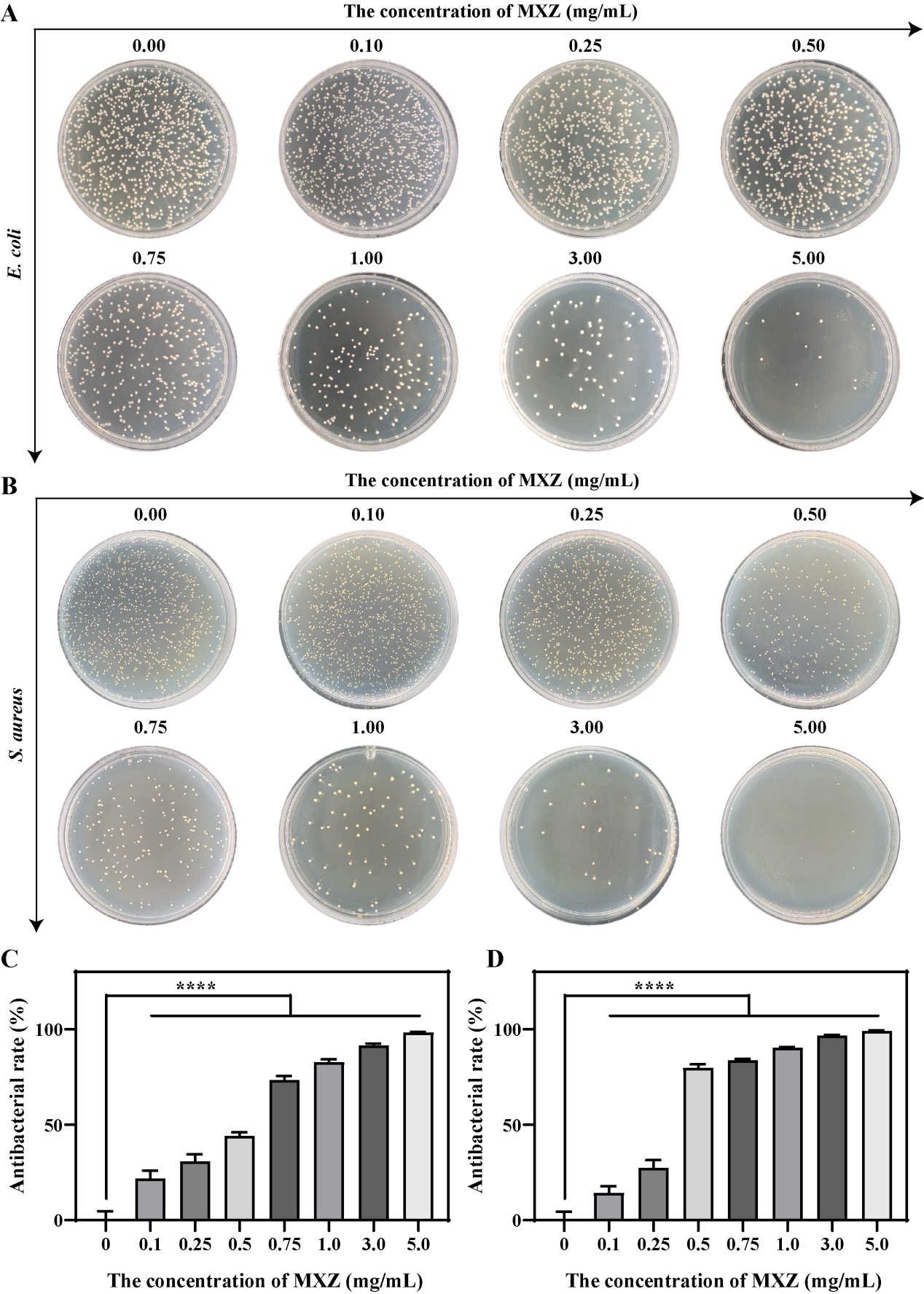


**Fig. S3.** Agar plate images of the antibacterial effect of MXZ at different concentrations against *E. coli* (A) and *S. aureus* (B); (C) Antibacterial rate of MXZ at different concentrations against *E. coli* (C) and *S. aureus* (D).


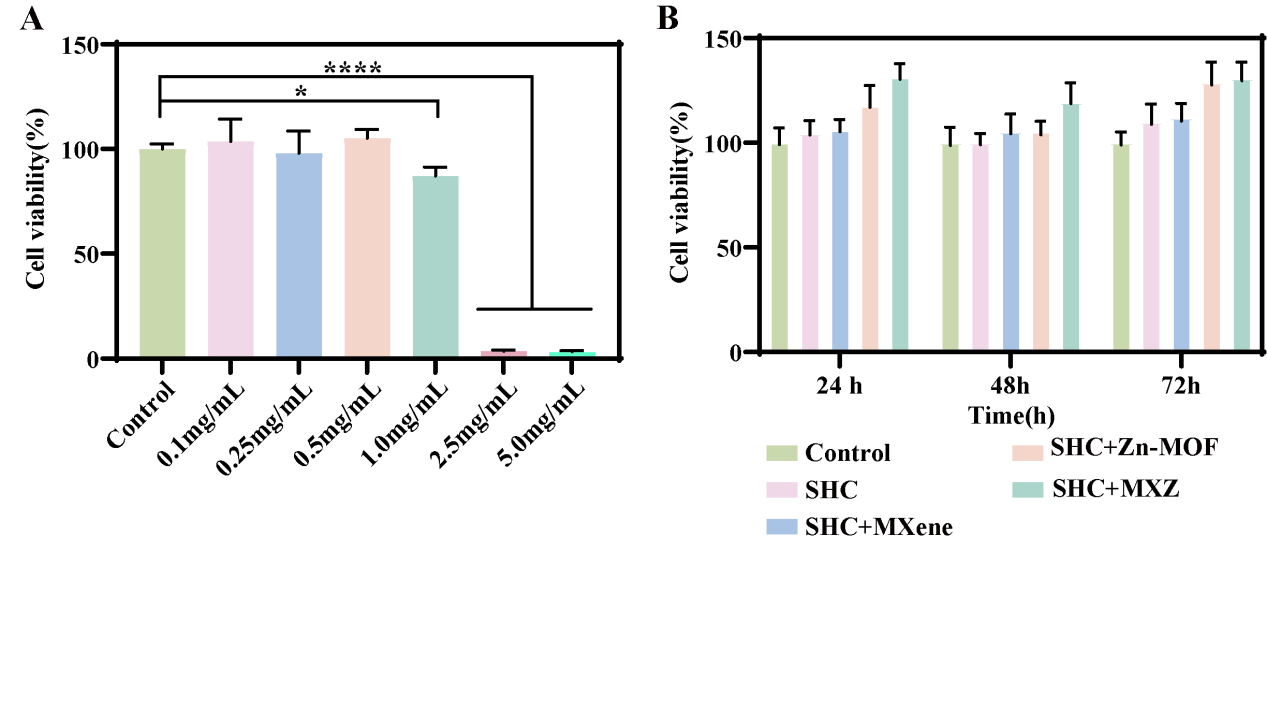


**Fig. S4.** A. Survival of cultured cells at different concentrations of MXZ as determined by CCK-8; B. Survival of cultured cells in different microneedles extracts as determined by CCK-8.


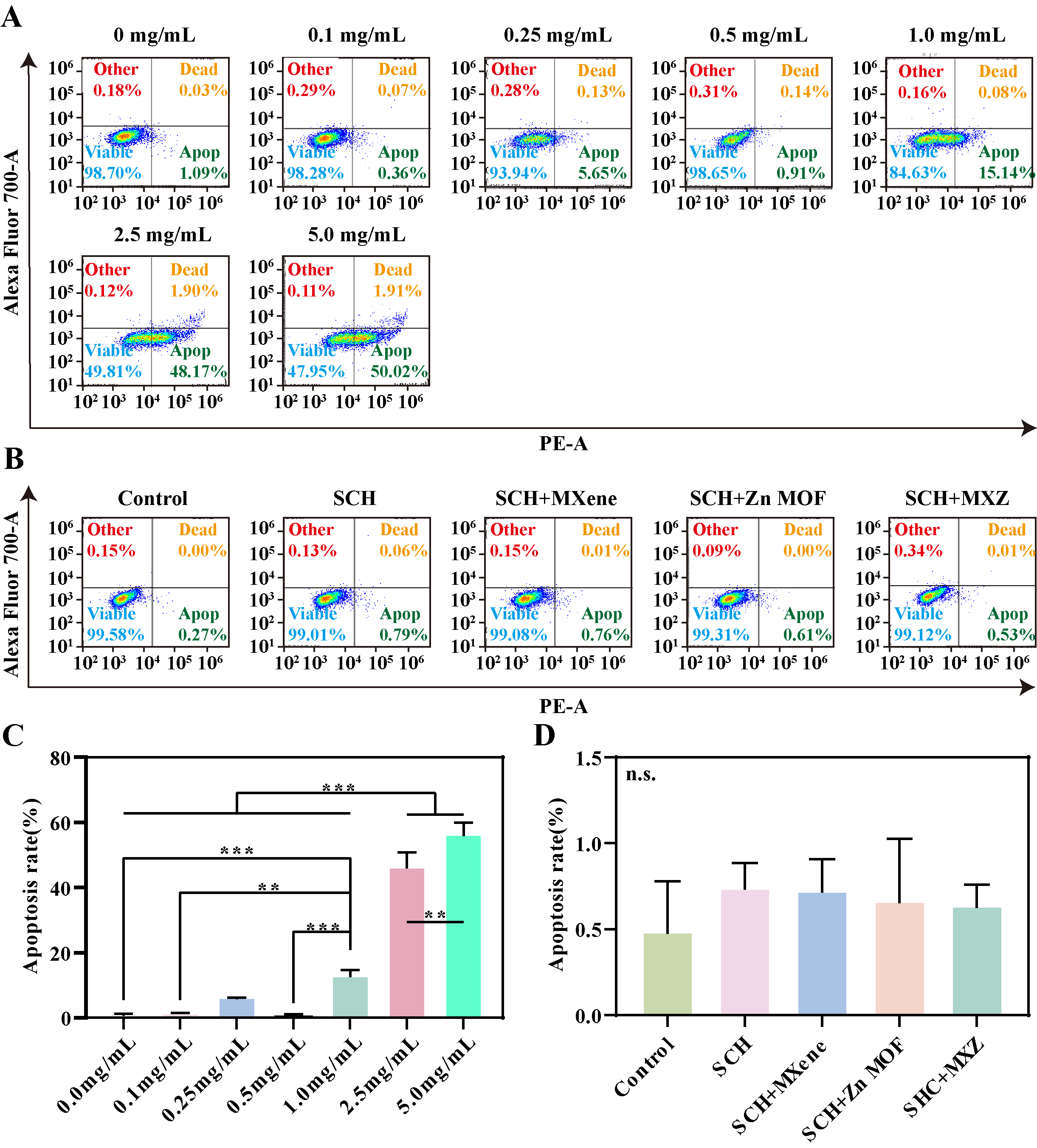


**Fig. S5.** (A) Flow cytometry apoptosis analysis of L929 cells treated with different concentrations of MXZ; (B) Flow cytometry apoptosis analysis of L929 cells treated with different microneedles; (C) Quantitative analysis of apoptosis in L929 cells treated with different concentrations of MXZ; (D) Quantitative analysis of apoptosis in L929 cells treated with different microneedles.


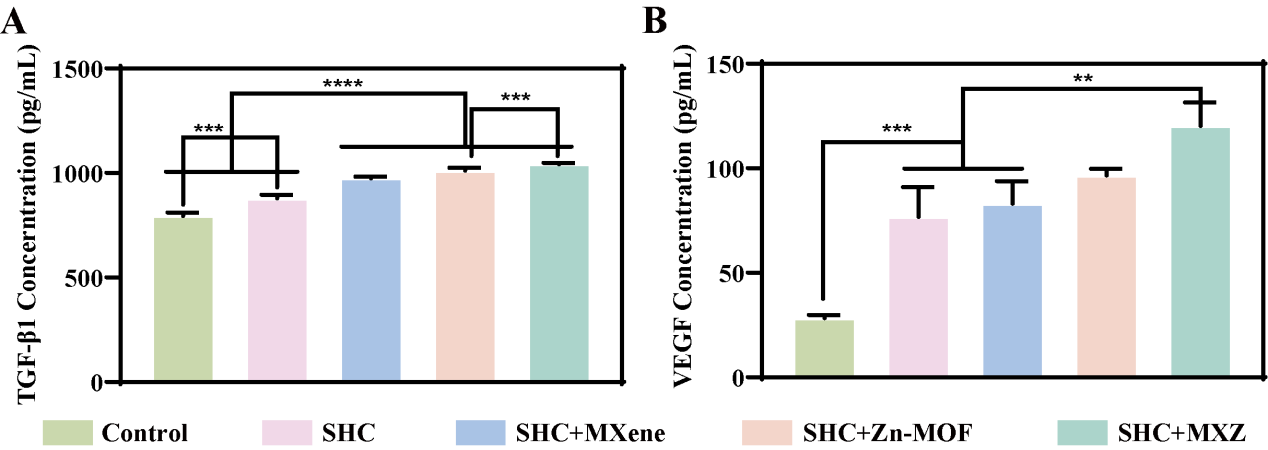


**Fig. S6.** A. The concentration of TGF-β1 in different groups by ELISA. B. The concentration of VEGF in different groups by ELISA.


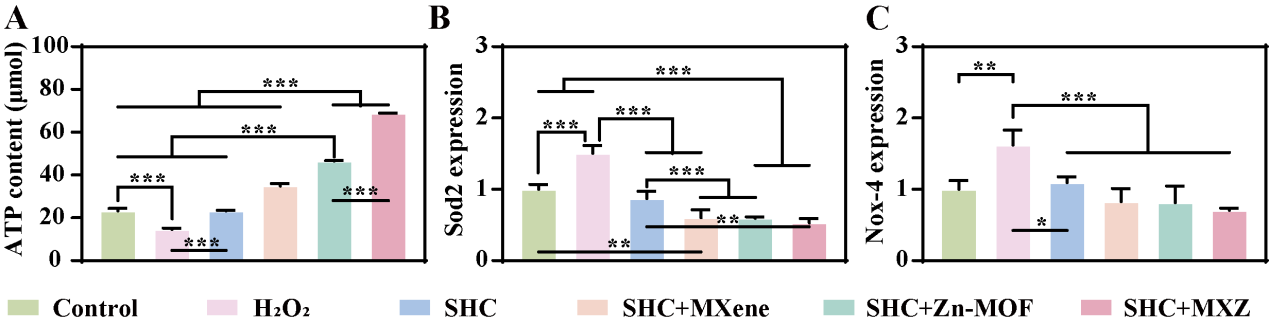


**Fig. S7.** (A) ATP levels in different microneedle groups; (B) qPCR analysis of SOD2 mRNA expression in different microneedle groups; (C) qPCR analysis of NOX-4 mRNA expression in different microneedle groups.


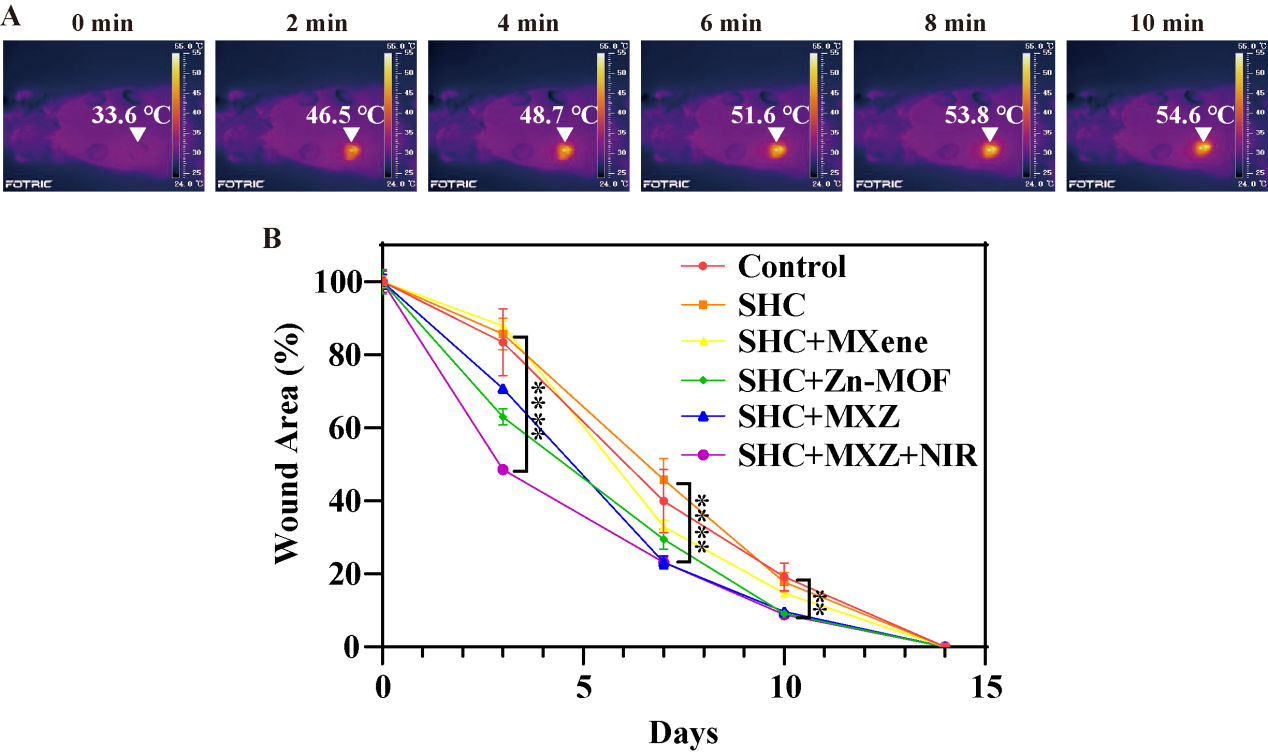


**Fig. S8.** A. Schematic diagram of the photothermal therapy process; B. Area of wounds at different times.

**
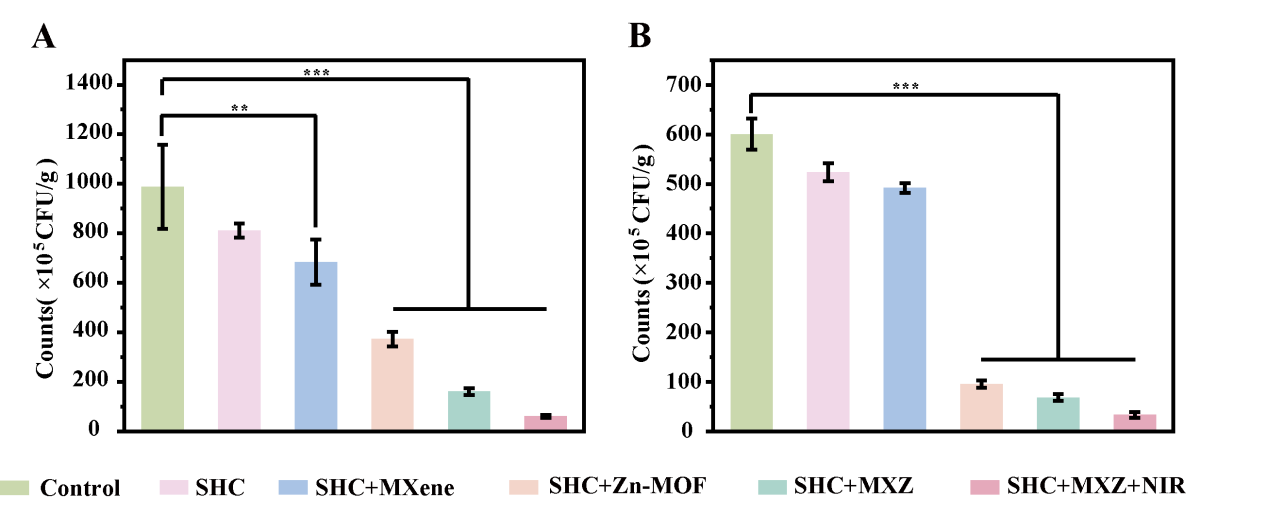
**

**Fig. S9.** A. Colony count of *E. coli* in wound tissue; B. Colony count of *S. aureus* in wound tissue.


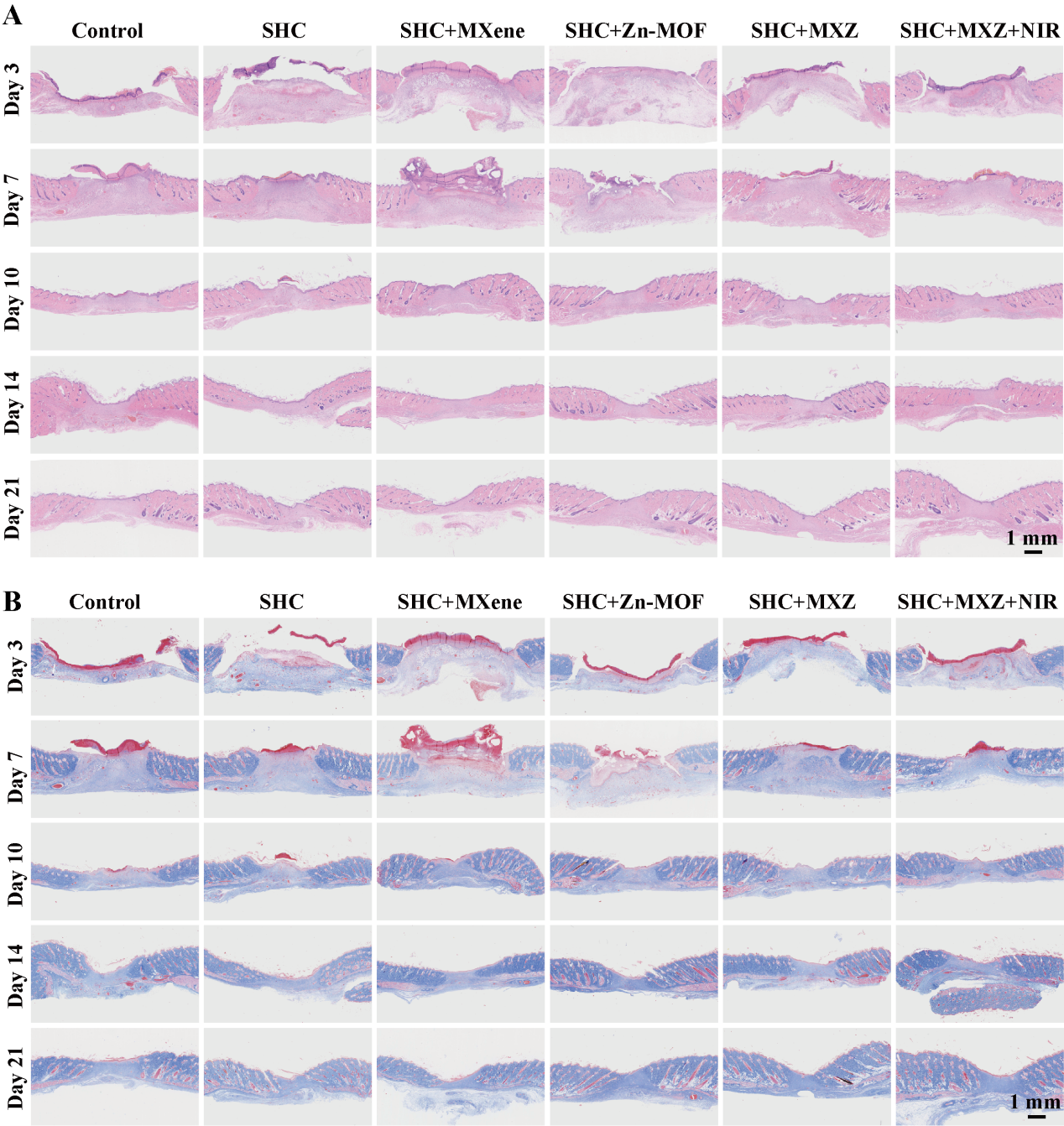


**Fig. S10.** H&E staining (A) and Masson staining (B) of the wound tissues.


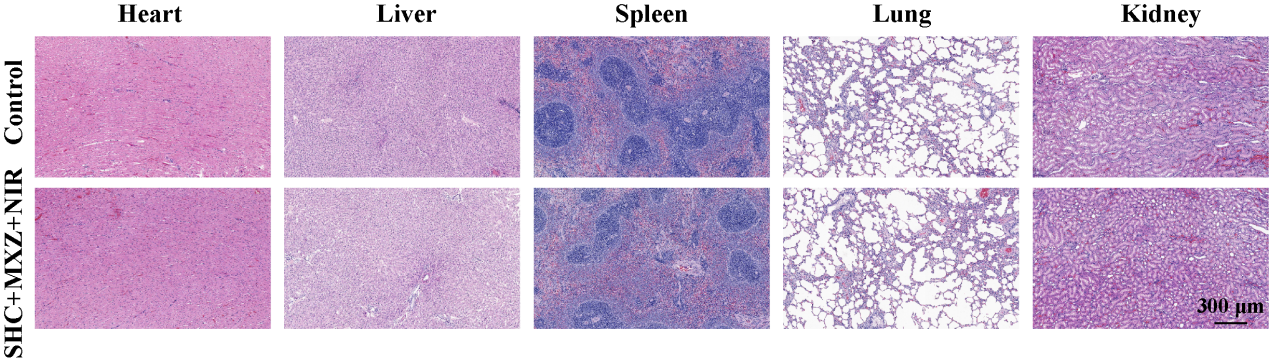


**Fig. S11.** H&E Staining of heart, liver, spleen, lung and kidney tissues in the control and SHC+MXZ+NIR group.


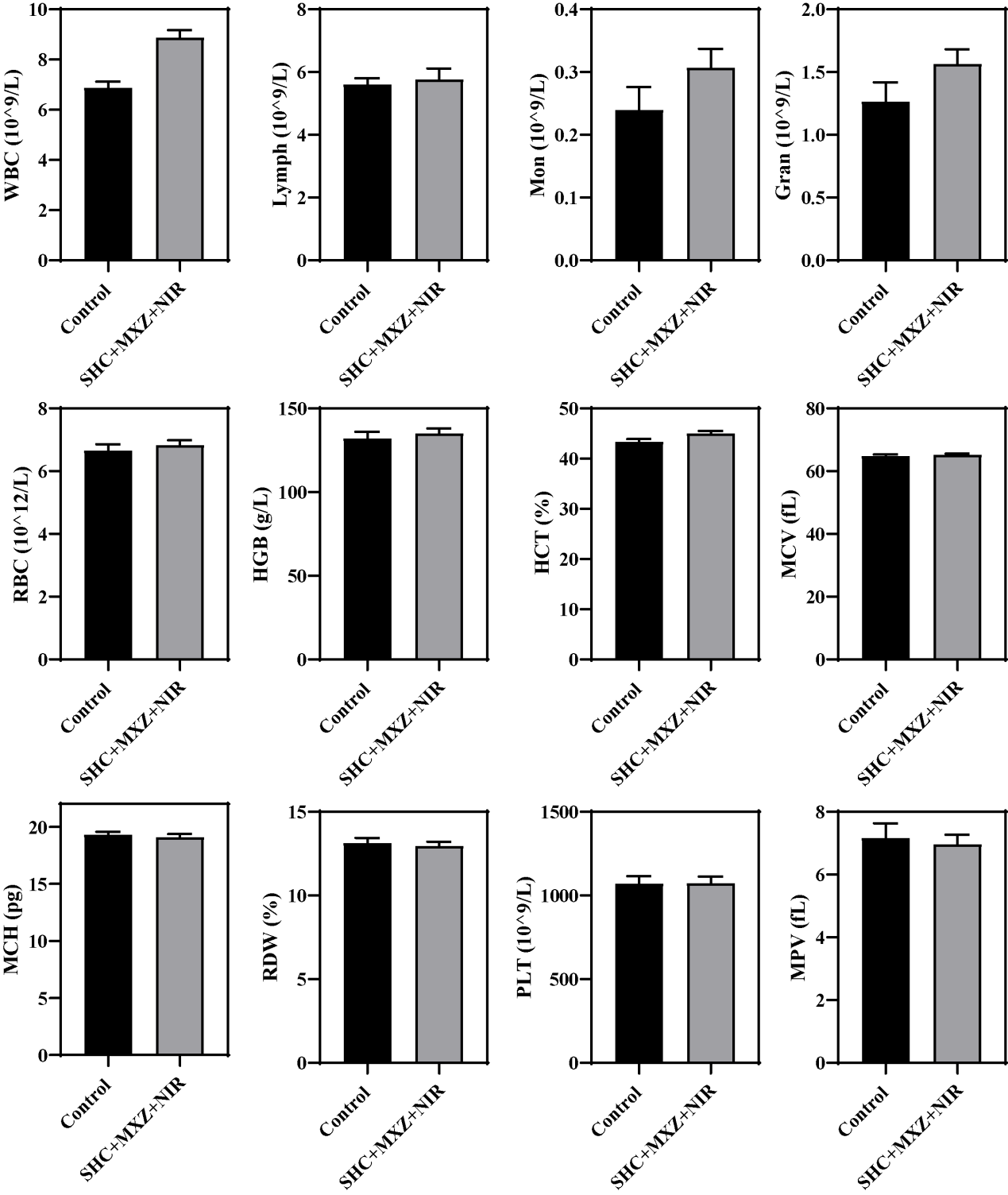


**Fig. S12.** Blood routine parameters of the SHC+MXZ+NIR group.


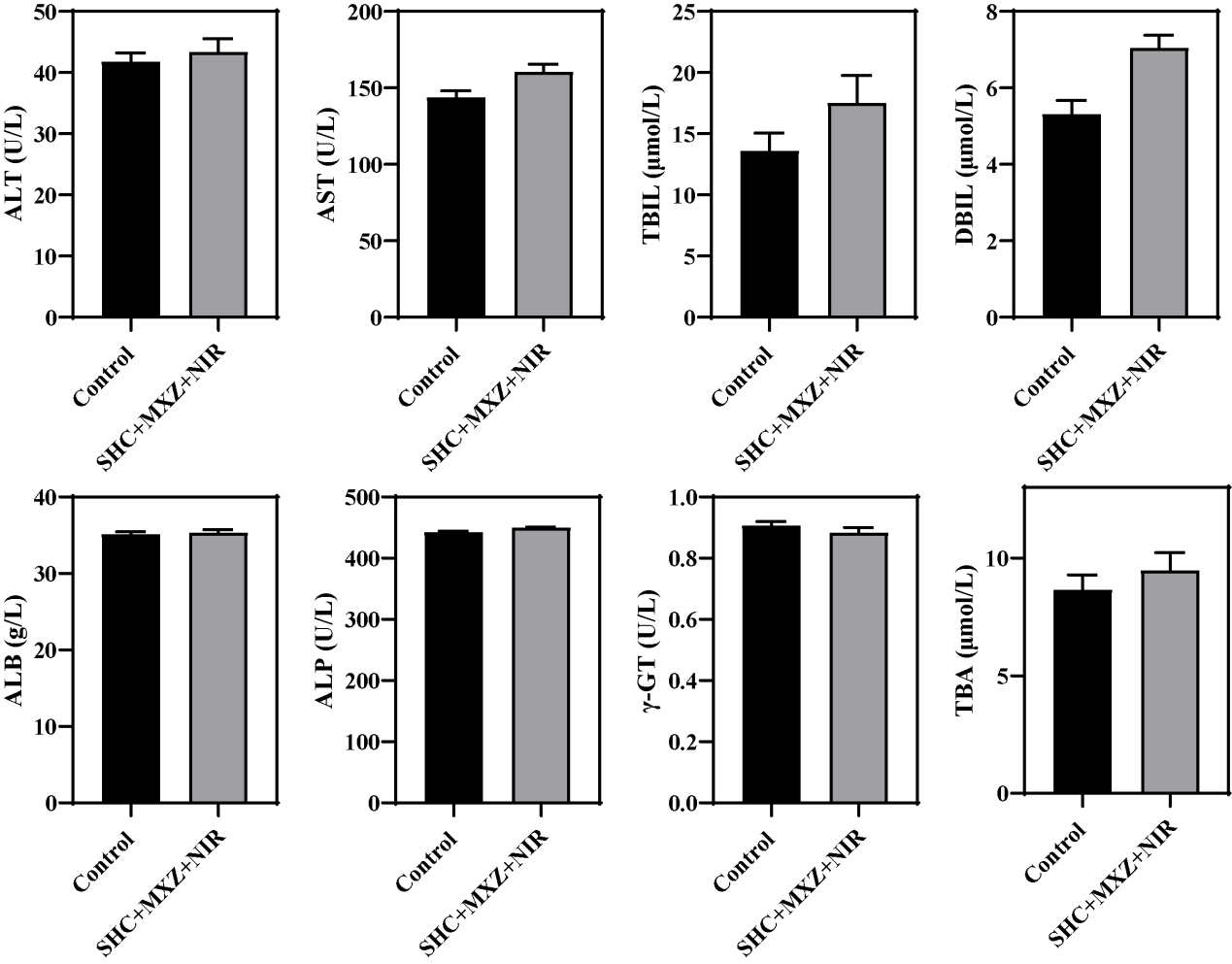


**Fig. S13.** Biochemical parameters of blood in the SHC+MXZ+NIR group.


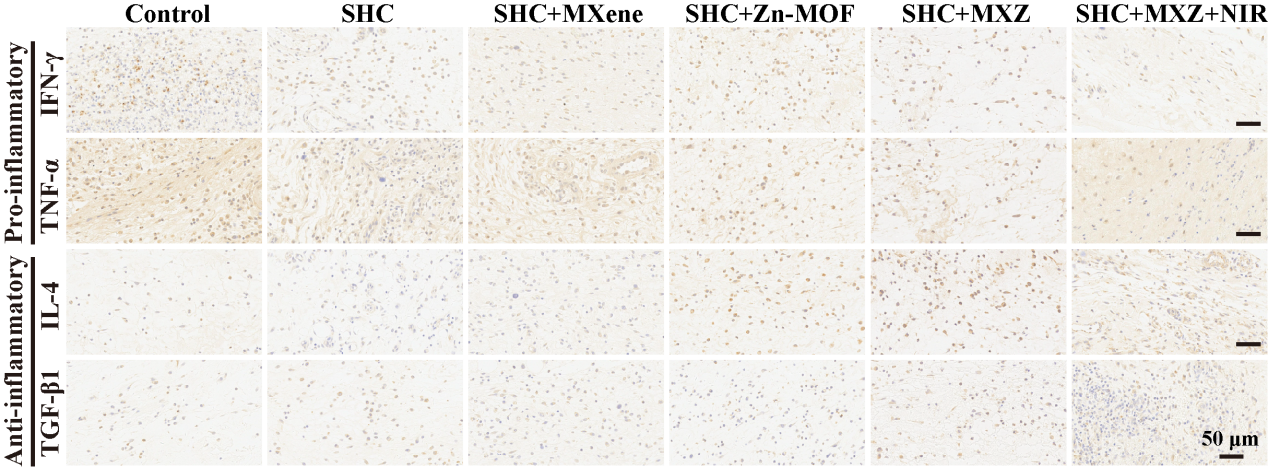


**Fig. S14.** Immunohistochemical staining of the wound tissues on day 3.


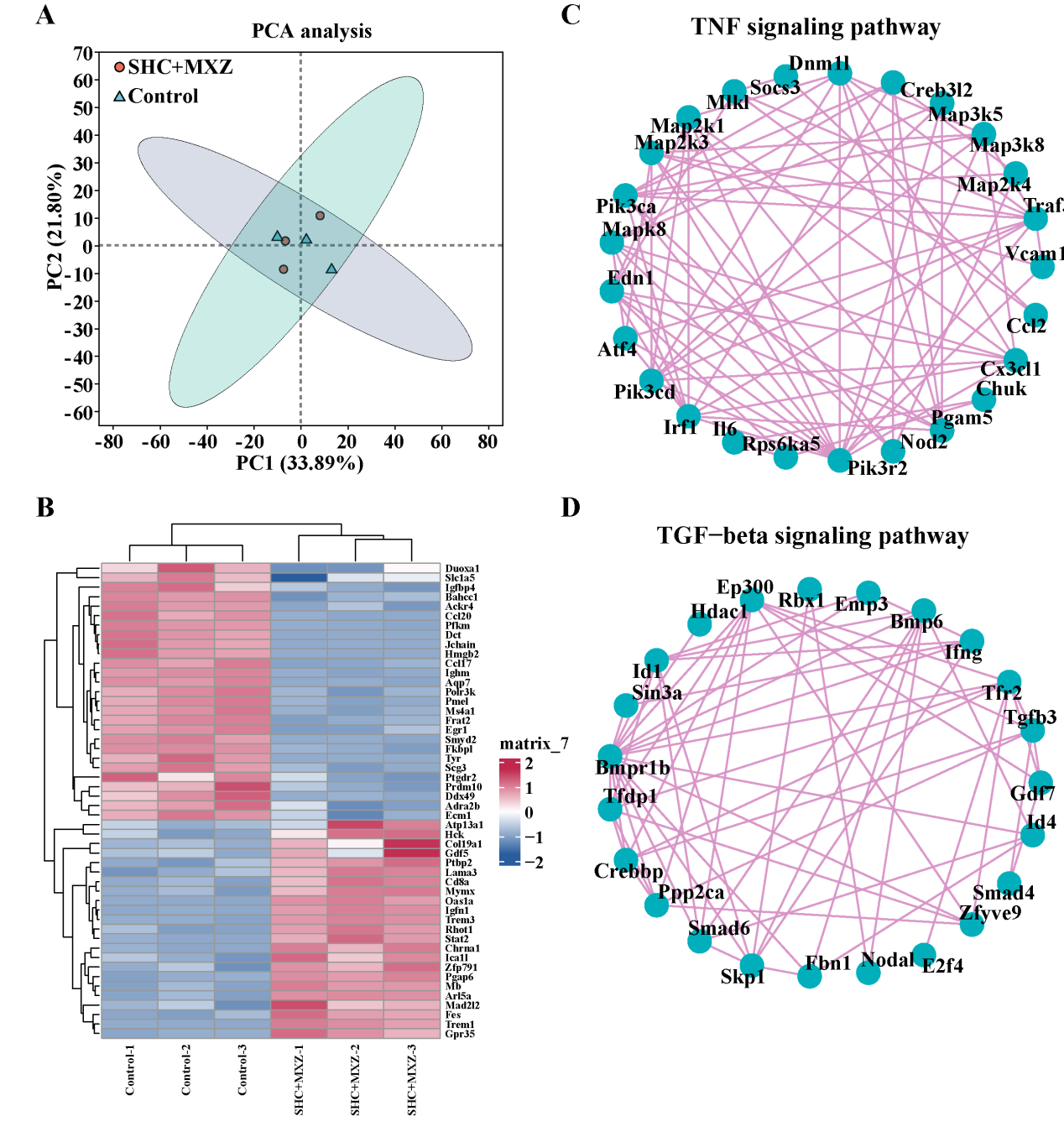


**Fig. S15.** A. PCA analysis of the control and SHC + MXZ groups; B. Gene expression heat map; C. Protein-protein interactions analysis of the TNF signaling pathway; D. Protein-protein interactions analysis of the TGF-β signaling pathway.
